# Digital Migration Infrastructure in return-writing: visualizing the migration landscape of India

**DOI:** 10.3389/fsoc.2024.1450773

**Published:** 2024-09-25

**Authors:** Preetha Mukherjee, Nirmala Menon

**Affiliations:** Digital Humanities and Publishing Studies Group, School of Humanities and Social Sciences, Indian Institute of Technology Indore, Indore, India

**Keywords:** digital, infrastructure, migration, return, homeland, refugee, conditional operation, ICT

## Abstract

Digitization has carved out the migration patterns of immigrants over the recent years of increased technological interventions in human mobility. Migration infrastructures, which typically refer to the physical, commercial, humanitarian, and governmental modes of operation, are multidimensional in nature. Digital infrastructures are equally important to the physical ones as digital technologies facilitate these migration processes through agents like hardware, software, and mediating actors. Amongst the multiple forms of migration, the concept of return-writing and nostalgia-struck-returnees encompass individuals whose life trajectories run parallel to the homeland. The narrative of return to the homeland emerges aβs a dominant motif in literature due to the rising trends of globalization, the writers’ reflection on their own migrant experiences, and publishing trends meeting the demand of the global book market. The objective is to assess the role of digital migration infrastructures in return migrations to India through a close reading of the selected texts and review of postcolonial literary theories by using conditional operation in Python. The study here explores the varied nuances of return migration with a primary focus on the external conditions of travel in migration literature. The paper aims to analyze the genre of return-writing in Indian English literature, through three novels over a period of two decades, i.e., from 2000–2023. The selected texts, beginning with Amit Chaudhuri’s *A New World* (2000), *Gun Island* (2019) by Amitav Ghosh, and Devika Rege’s novel *Quarterlife* (2023), offer a panoramic view of return migration. These novels are extensive in the time period of technological interventions and in depiction of return migration. The Python code examines the extent of existence of a set of digital migration infrastructure keywords by analyzing the content of the novels and creates bar plots and charts to offer a visual representation of the classification results. The resulting trend traces the increased intervention of Digital Migration Infrastructure in the recent migration literature.

## 1 Introduction

[M]igration has always been, since the very beginning, central to the human story. And remains so—(Miller, 2023).

Migration has defined the postcolonial condition of human mobility. Though, historically and culturally, migration forms the fundamental element of the human story, “borders and passports and quotas and walls and visas” are increasingly defining our “identity, ethnicity, religion, idea of home, patriotism, nostalgia, integration, multiculturalism, safety, terrorism, racism.” Miller (2023) goes on to say that the word “migrant” “clumps together people whose experience of migration is extremely diverse, slaves and spouses, refugees and retirees, nomads and expats, conquerors and jobseekers. It’s a hypernym, an umbrella term which can be used to cover those who intended to migrate.” It also refers to those who decided to stay back and those who decided to return to their country of origin.

The literary representations of migration have been dynamic in its multiple forms, its heterogeneous migrant experiences and its duality of physical and digital infrastructures have evolved over the years. Digitization has carved out the migration patterns of immigrants over the recent years of increased technological interventions in human mobility. Migration infrastructures, which typically refer to the physical, commercial, humanitarian, and governmental modes of operation, are multidimensional in nature. Digital infrastructures are equally important to the physical ones as digital technologies facilitate these migration processes through agents like hardware, software, and mediating actors. “As Migration Infrastructures are defined as organized structures, Digital Migration Infrastructures primarily encompass the overarching structure that enables virtual communication. Digitization has carved out the migration patterns of immigrants over the recent years of increased technological interventions in human mobility. Migration infrastructures, which typically refer to the physical, commercial, humanitarian, and governmental modes of operation, are multidimensional in nature. Digital infrastructures are equally important to the physical ones as digital technologies facilitate these migration processes through agents like hardware, software, and mediating actors. “As Migration Infrastructures are defined as organized structures, Digital Migration Infrastructures primarily encompass the overarching structure which enables virtual communication.” Digital Migration Infrastructure (DMI) hence defines the distinct role of digital media technologies in migration and identifies the use of ICT involved in its entailing process. The studies on DMI are sparse and primarily focused on forced displacement based on an empirical analysis. The role of DMI in postcolonial literature, in the genre of return-writing, has largely been absent. The objective is to assess the role of digital migration infrastructures in return migrations to India through a close reading of the selected texts and a review of postcolonial literary theories by using conditional operation in Python.

Indians have been immigrating across the world, as early as 15th century with traces of Indian. colonies in Indonesia (Jain, 1989), building settlement and diaspora communities in various places. The data of Indian immigrants have been made available at the public platform from 1970s, that started from a population of about 3.4 million people (Jain, 1982). Indian immigrants as also discussed by Madhavan (1985) typically consist of people scattered across due to economic expectations, jobs, research, education, marriage or as indentured labors during colonization. The multicultural and multilingual element becomes the characterizing element of the immigrants. India is not a single cultured society with one dominant language but rather a largely diverse land with motley religious practices, traditions, culture and food habits as represented in the immigrant community. It is also characterized by assimilationist tendencies, holding strongly to the collective community consciousness. These immigrants time and again return to their homeland for routine visits or permanent return in some cases. Return writing or the narratives of return, though a frequent instance in postcolonial literature is not widely acknowledged as a dominant genre. The concept of return-writing and nostalgia-struck-returnees encompass individuals whose life trajectories run parallel to the homeland. Return writing “seeks to remedy widely held anxieties about cultural loss and the erasure of personal and family histories from public memory.” The genre of return writing reverses the migrating subject to the new Indian city that often tends to cast the individual as an outsider. The “narrative of return” (Thompson, 2014) to the homeland emerges as a dominant motif in literature due to the rising trends of globalization, the writers’ reflection on their own migrant experiences, and publishing trends meeting the demand of the global book market. In return writing, the return is projected in time and space with a clear depiction of the returnee and the individual’s condition of return. The returnee resides in a state of hybridity, rooted in two or more places of belonging. However, return migration is largely heterogeneous in its literary representations and can be identified in various forms depending upon the period of stay and return. The tropes of return range from actual to symbolic returns, provisional to permanent returns and diasporic visits, which we will analyze in depth in the context of the novels in discussion. Return, not necessarily a voluntary act, requires ascertaining resource mobilization and returnee’s preparedness, which varies for every migrant. The study here explores the varied nuances of return migration with a primary focus on the external conditions of travel in migration literature.

The paper aims to analyze the genre of return writing in Indian English literature through three. major novels over a period of two decades, i.e., from 2000–2023. The selected texts, beginning with Amit Chaudhuri’s A New World (2000), Gun Island (2019) by Amitav Ghosh, and Devika Rege’s debut novel Quarterlife (2023), offer a panoramic view of return migration. These novels are extensive in the time period of technological interventions and in the depiction of return migration. The Python code examines the extent of existence of a set of DMI and ICT keywords like “computer,” “telephone,” “mobile,” “internet,” “money transfer”, “online”, etc. by analyzing the content of the novels and creates bar plots and charts to offer a visual representation of the classification results. The research objectives extend beyond the descriptive analysis of the novel to highlight the experiences of Indian immigrant on return and the return intentions framing the nature of return through literary representations and how the digital infrastructure plays a dominant role in it.

## 2 Migration and the digital intervention: Digital Migration Infrastructure

[I]nfrastructural approach addresses migration as a constellation of non-migrants and migrants and of humans and non-human actors—(Leurs, 2020).

As a “black-boxed”^1^ invisible apparatus, infrastructure functions in the background at the base of any operation. It is inherently embedded in a country’s socio-cultural, economic and political relations. “To study infrastructure is, in truth, to study the technologies and techniques through which the visible and invisible are separated, connected and managed in the social life of cities” (Appadurai, 2015). The infrastructural establishments are often “standardized repeatable phenomenon” (Bingham et al., 2023) and transcend beyond the urban centers of cities to the far remote corners, facilitating a network of exchange and communication to aid progress and development for mankind. Larkin (2013) defines infrastructures as “built networks that facilitate the flow of goods, people, or ideas and allow for their exchange over space. As physical forms, they shape a network’s nature, its movement’s speed and direction, its temporalities, and its vulnerability to breakdown.” Infrastructure as a physical dimension carves out human mobility and migration. As the term suggests, migration infrastructure refers to organizational structures that determine how migration takes place, the use of resources to migrate, and the key elements of migration aspiration. It plays a significant role in migration across continents in ascertaining the systematic movement of people as “mobilites cannot be described without attention to the necessary spatial, infrastructural and institutional moorings that configure and enable” (Hannam et al., 2006). Migration infrastructure serves as the backbone of the migration industry which derives profit from serving the migrants. The five dimensions of migration infrastructure are “interrelated” (Xiang and Lindquist, 2014) and range from commercial, regulatory, humanitarian, social and technological, which encompass recruitment agencies, documentation and training, NGOs, migrant networks and communication, respectively. As a migration industry^2^, it is a configuration of all factors that facilitate and institutionalize migration. The term, first used by Cohen (1997), encompasses the direct and indirect agencies that intervene in migration processes, such as travel agencies, lawyers, recruiters and brokers. However, though a part of the migration industry, migration infrastructure differs from it in facilitating migration rather than preventing it (Leon, 2013). It refers to the “institutions, networks and people that move migrants from one point to another” (Lindquist et al., 2012). Düvell and Preiss (2022) discuss the three major types of migration infrastructure as categorized Table 1, Migration Infrastructure Typology. Technology and digital infrastructure shape the current digital age. Hence, migration infrastructures emerge as “multidimensional’ in the symbiosis of “nature and technology, structure and agency and knowledge”. It is at the same time important to note that these digital infrastructures are a result of human effort, of a set of individuals who have envisioned and curated this infrastructure. It is not a disembodied digital experience but rather encapsulates the human experience that goes into its making. Press goes on to comment that “digitization has fundamentally shaped the way people migrate over the last years” (2022). Digital platforms offer multiple digital resources to enable a smooth interaction between humans and technology. Since our primary area of study is digital migration infrastructure, we will investigate the third typology, i.e., the intervention of technology and digital migration.

**Table 1:** Migration infrastructure typology.

Category	Types	Examples
I	Actors in migration infrastructure	Private and government agencies
II	Material Migration Infrastructure	Transportation and transit migration hubs
III	Digital migration infrastructure	Hardware, software, and actors

Source—introduction to migration studies.

Digital Migration Infrastructures (DMI) seek to address the role of digital media technologies in migration. Though a set of technologies and digital platforms (Constantinides et al., 2018) form the digital infrastructure, “What distinguishes infrastructures from technologies is that they are objects that create the grounds on which other objects operate, and when they do, they operate as systems” (Larkin, 2013). It enables migration processes and encompasses virtual communication across borders. “Digital migration infrastructures are the ensemble of digital technologies including the underlying support structures which facilitate migration processes” (Press, 2022). The use of such infrastructure determines the use of digital media and technologies during the whole process of migration from origin to destination. DMI is as important as the physical infrastructure, and digital technologies cannot be considered as “inseparable from offline material”^3^, as they emerge from historical, socio-political contextual dynamics. The existing literature on DMI focuses on country-specific forced displacements and “selfie-taking refugees”. Our study on DMI focuses on how the various digital mediums and ICTs support the international migration processes and their role in maintaining virtual communication with kin at home. The predominant area of migration in the study is the literary narratives of return migrants, which will be discussed in the following sections.

Leurs (2020) states that the technological infrastructural innovations in migration are deeply. hierarchical and shaped by “dislocation, contestation, hybridity, mimicry and adaptation.” In discussing the manifold forms of digital migration infrastructures, Press (2022) distinguishes the ICTs into three broad categories of “hardware”, “software” and “actors”, as digital technologies drive migrants into smoother immigration or emigration. These infrastructural ICTs are codependent and cannot function independently without each other (Leurs and Smets, 2018). For instance, a smartphone cannot be put to use without a working network connection offered by a service provider. The harmonious relationship between the hardware, software and actors results in a seamless route and in-transit experience for the migrant. At the same time, a migrant’s digital presence and communication is hierarchical in nature, characterized by conflicts within groups and communities. An investigation into the “sociodemographic profiles" (Carames et al., 2021) and migrant projects reveals that digital platforms create virtual communities as social spaces of interaction in an online environment without the need to invest any emotional resources. However, this digital presence functions in a hierarchy in its integration of migrant’s social inclusion and exclusion, depending largely on the source country, the destination and their past experiences of migration.

**Figure 1:**
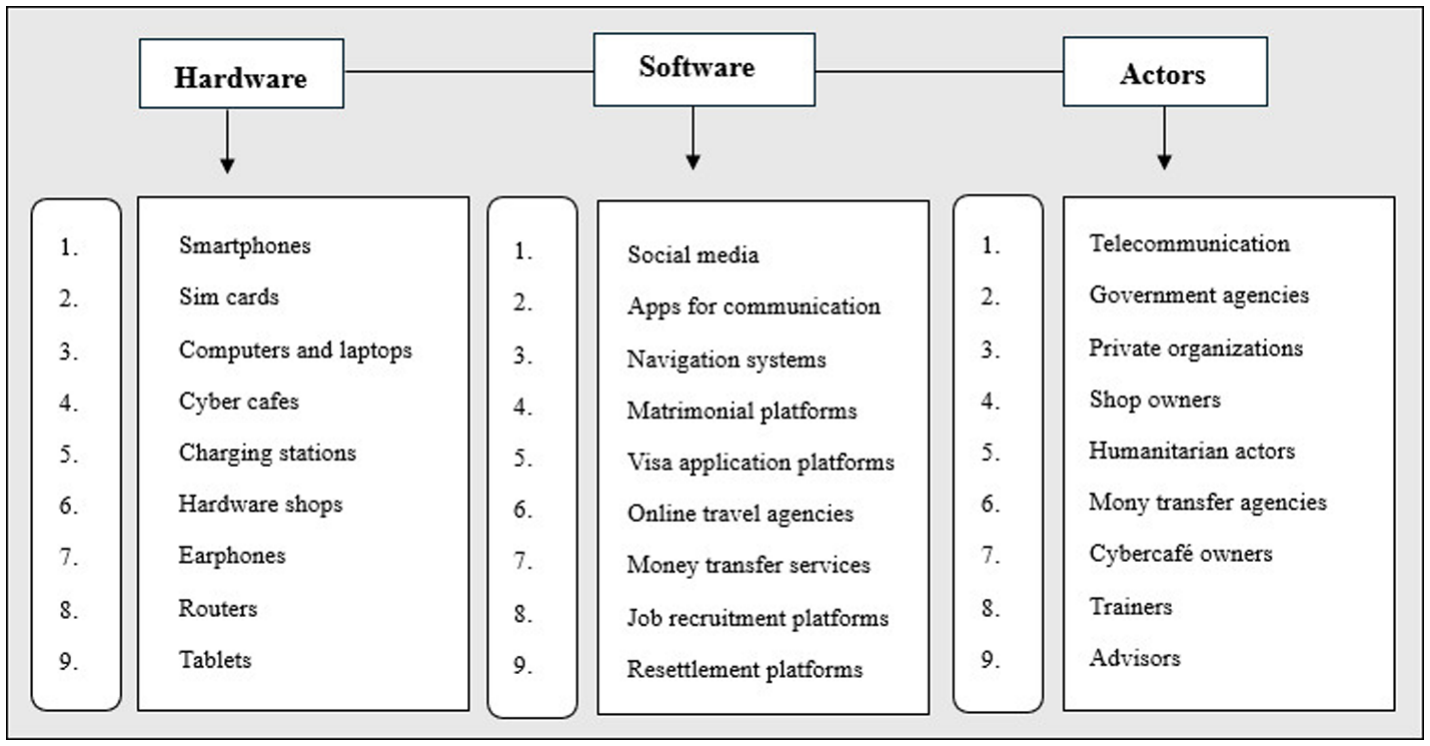
Digital Migration Infrastructures.

In reference to Figure 1, Digital Migration Infrastructures, the dominant ICTs in migration are smartphones and telecommunication, around which the digital ecosystem is built. In tracing migrant journeys, studies have revealed that most migrants are equipped with at least one digital device that connects them to places across the borders and allows them to navigate in a digitized environment. Diminescu (2008) first proposed this idea of a “connected migrant” with a “connected presence which first manifests itself through permanent accessibility. This is composed of different forms of potential or actualized presences whose common characteristic is that they are never complete, as is the case in face-to-face presence.” Though connected migrants are endowed with access to capital like a smartphone to connect to their territory of origin, they are mostly absent from there. ICTs intervene to mold the incomplete absence and presence to attain a remote presence but rather results in an absence presence. This brings us to Sayad’s (2018) notion of “double absence”, the spatial relation of a migrant to both source and destination regions, giving into the absence while trying to be present at both locations. If the ICTs, which primarily aim to build a virtual connection with the migrants, dwell in a precarious absence, it becomes important to note the advantages and limitations of their usage. Technology is pharmacological^4^, an analogy between medicine and technology that highlights both digital innovations’ curative and destructive power. “[A]ll technology is originally and irreducibly ambivalent” (Diminescu, 2008), and this ambivalent nature arises from the increasing dependence on technology. Positive pharmacology meets the pressing needs of the migrants in the migration process and marks their virtual presence, but at the same time, it brings about a sense of absence within the migrant. Other negative factors pertain to meeting the expectation of family and friends to be in constant contact and provide quick financial aid.

Mille (2013) proposes a new model to analyze and trace migration called the migration traceability to document the trajectory of migration through digital prints like a logbook. It consists of the digital traces of a migrant, which he or she leaves behind at various points of their journey, creating a digital environment. “The digital trace consists of the digital prints left accidentally or on purpose in the IT environment during an IT process.” In contrast to migration before the digital age, it serves as a story of personal information that needs high automatic processing to be decoded. Following both a quantitative and qualitative approach, migration traceability adapts the methodology of digital humanities, intersecting both statistical data and life narratives. As Diminescu (2020) describes, migration traceability reconstitutes the migratory itinerary in real time, spanning over long periods as digitization of personal archives of migrant experiences.

### 2.1 Information and communication technology (ICT) as the core of Digital Migration Infrastructure (DMI)

“ICTs generate qualitatively new ways of living together at a distance in migration contexts. ICT-mediated practices provided the premises for a transnational everyday reality to emerge, which is based on ubiquity, simultaneity and immediacy of interaction over borders” (Baldassar et al., 2016). In this section, we will focus on how DMI, through ICTs, plays a significant role in transnational communication after the migration takes place. Migrants employ various ICTs to mediate their multiple cultural belongings and migrant identity. Baldassar depicts the types of co-presences that a transnational migrant may experience in his or her interaction with ICT to maintain types across borders.

As shown in Table 2, Transnational Co-presences with and without ICT, we can see how ICT facilitates only a virtual co-presence with the kin in the homeland. However, Baldassar et al. (2016) talks about a new “ambient co-presence facilitated by polymedia environments”. The interaction between digital platforms and multiple ICTs creates an “intense yet peripheral experience” of an “always on” culture, the awareness of a constant presence of a face-to-face co-presence through social networking sites.

**Table 2:** Transnational co-presences with and without ICT.

Sl No.	Types	Description	Examples
1.	*Physical*	The actual physical presence in the longed-for place with the longed-for person.	In person meetings
2.	*Virtual*	Communication through sense of hearing or seeing by video calling, verbal exchanges over mobile or though written messages.	Phone calls, text or audio messages, video calls through social media platforms.
3.	*Proxy*	Contact through transnational objects or an individual whose physical presence embodies the spirit of the longed-for absent place or person.	Photographs, memorial objects, fellow friends from the same migrant community, visiting family.
4.	*Imagined*	Establishing a sense of contact and togetherness by imagining a co-presence when in reality, they are not engaged in any active communication.	The act of remembering family in daily prayers.

Source from Baldassar et al. (2016).

### 2.2 Selection of Digital Migration Infrastructure Keywords

**Figure 2:**
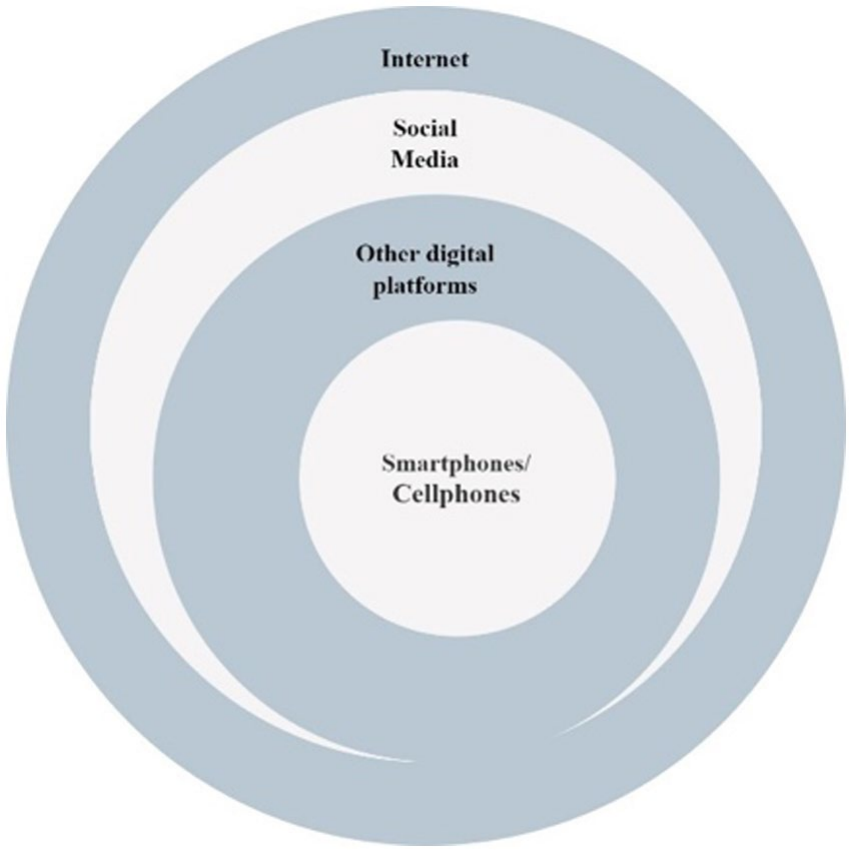
Keywords.

As depicted in Figure 1, the interplay of multiple digital infrastructures under the broad categories of hardware, software and actors enables a hassle-free migration experience. However, for the need for our study here, we will be selecting a few major ICTs involved in the process that are relevant to our selection of the literary texts. These ICTs or digital devices directly or indirectly give access to navigation and communication of the migrant in a digitized environment. “Digital passages”^5^ are an embodiment of these infrastructural facilities, which frame the migrant identity across the digital space. The passages often serve as a guide to human traffickers, smugglers and agents who use the routes for illegal migration across borders. These passages encompass internet access, social media, Wi-Fi zones in transit, charging stations, GPS and other ICTs. As “accessing crucial information on the Internet depends on an entire infrastructure,” “the reliance on both infrastructure and artefacts affects the interplay of some of the major protagonists in this sociotechnical space: refugees, smugglers, corporations and government” (Latonero and Kift, 2018). Technology hence meets all the crucial needs for the journey of migrants. The digital platform endowed with the migration infrastructure serves as the “habitus”^6^, where migrant communities engage in digital practices that become the source of their habituated disposition. It generates a new transnational habitus for migrants across different spaces of time and place. The habituated routine across the digital platforms provides the migrants with a sense of assurance and stability after they have settled at the migrated destination. Hence, the selection of keywords depends on the more commonly accessed ICTs and readily available digital devices as visualized in Figure 2.

Smartphones have become a dominant part of DMI. Smartphones are portable, affordable and user-friendly as digital devices for establishing communication, entertainment, navigation, planning and documenting the journey. Leurs and Patterson (2020) discuss the three major roles of smartphones. Migrants, as displaced individuals, make effective use of smartphones for survival, transnational communication and emotion management and digital self-representation. “Smartphone traveling”^7^ plays a pivotal role in accessing information during the whole process of migration, establishing transnational connectivity and assisting in mapping journeys. The intersection of smartphones and social media enables smart journeys based on shared data location on social networks. As portable archives of past and present, through selfies, written posts, videos, messages and recordings, they portray the two sides of migrant lives, the horrid realities of refugees and asylum seekers and the glorious diaspora of the privileged migrants. In the post-migration scenario, social media comes in handy to understand the region’s cultural and social nuances. It serves the need to meet the language demands, cultural ideas and societal expectations. “Multiple modalities of social media apps may increase feelings of belonging and emotional well-being among displaced migrants and their families” (Leurs and Patterson, 2020). The Internet provides access to social media and other digital platforms like travel agencies, matrimonial, money transfers, and other applications on smartphones, laptops, tablets, and computers. Hence, the selected keywords are all interrelated in their effective functioning as DMI. “ICTs have been identified as key issues in migration. However, there is a lack of knowledge about how the use of ICT exactly impacts the way that migration wors” (Zijlstra and Liempt, 2017). The study aims to unravel the use of ICTs as part of digital migration infrastructure in the journey and migrant lives of individuals in literary reflections of return migrants in five selected Indian English novels from 2000–2023.

## 3 Conceptualizing return migration

The newly emerging figure of a migrant substitutes the paradigmatic figure of a migrant as displaced from his native land and uprooted from his native culture. The contemporary notion of voluntary migration to seek better opportunities overseas lies in stark contrast to the notion of forced migration, exile and refugee movements, where return brings back the trauma of rampaged movements, political chaos and dilapidated houses. Migrant mobility between places of origin and destination characterizes their transnational way of life. The idea of return migration or returning back home is a glorious affair mostly for voluntary immigrants and whose return is celebrated back in the homeland. “The question of returning home, to the homeland, the country of origin, the place left behind is intrinsically linked to the migration decision and how the migration trajectory ultimately is experienced” (Baas, 2015). Returns for multiple purposes is becoming more common due to advanced digital migration infrastructures, affordable transport and easy journeys. In this section, we will study return as an emerging concept of migration studies and analyze the various causes and situations of return in the context of existing socio-economic theories.

“Return migration is defined as the movement of emigrants back to their homelands to resettle” (Gmelch, 1980). Traditionally conceptualized as a return to base or home, return migration mainly refers to the permanent journey back home. However, on tracing the migratory patterns, we can see that the trajectory of return varies from one migrant to the other and always does not necessarily mean a permanent return intending to resettle, which fabricates a transnational identity. The transnational identity as a synergy between identities acquired both in source and host countries are not in conflict with each other. Rather, it leads to the construction of double identities, which the migrants switch depending on their location. However, the hybridity persists within the ambiguity of identities as a typical characteristic portrayed in migration literature. “Today’s self-proclaimed mobile and multiple identities may be a marker not of contemporary social fluidity and dispossession but of a new stability, self-assurance and quietism” (Young, 1995). Postcoloniality remains (Young, 2012) and so do postcolonial migrant figures who are torn between two or more cultures in asserting their identity, which is constitutive of the hybridization of national and transnational forms severed from an “essentialized nativist identity” of the homeland. This hybridity constructs a liminal and dialogic space wherein the migrant identity is negotiated. The idea of hybridity today is more of a “more celebratory pickle capable of disrupting binaries of the east–west/ colonized-colonizer” (Menon, 2016). Postcolonial migration has disrupted the extreme binaries of the East and West, as the peripheral existence comes closer to the center until the center becomes a part of the periphery through the model of return migration.

The types of return and return intentions are both interlinked. “Return motives can be highly diverse, ranging from socio-economic exclusion in the country of destination to successful business investments” (Haas De et al., 2014) leading to either return of success or failure. The deciding factors of return often range from economic conditions, social ties, occupation and education, duration of stay and past migration experiences. The Neoclassical (NE) model suggests migration for profit maximization contrasted against the Neoclassical Economics of Labor (NELM) model where the migrant returns on achieving the desired goal of profit. Cassarino (2004) also discusses that the NELM model serves as a “calculated strategy” in deciding the outcome of the migration. The level of preparedness also decides the migrant’s readiness to return and at the same time depends on resource mobilization. “The returnee’s preparedness refers to a voluntary act that must be supported by the gathering of sufficient resources and information about post-return conditions at home” (Cassarino, 2004). Accordingly, he distinguishes three levels of preparedness which ascertain the return. Returnees with a high level of preparedness have time to analyze the economic costs and benefits ensuring a smooth return. Returnees with low levels of preparedness are those migrants who have stayed abroad for a shorter duration, allowing limited resource mobilization which makes their cost of staying more than that of return. However, there are some returnees whose level of preparedness is altogether null. Their return is abrupt without any plan or earlier intention with no preparation for the return. The level of preparedness largely depends on resources available at hand and the circumstances in both host and home countries. Economic crisis hence frames the return intentions for a short term. Parella and Petroff (2019) in this context refers to Faist and King’s model of double embeddedness built on the multiple levels of macro-meso and micro approaches. This in turn gives rise to three profiles of returnees resulting in unplanned return or stay, of premature return, return as a disruption and forced stay due to lack of resources to return. The economic conditions of the returnee hence play a vital role in deciding the duration and intention of return. The novels discussed in the following sections portray returnees with a strong financial support which makes their return easier. Parella et al. (2019) also goes on to suggest a “conceptual framework” where social suffering impacts the return of migrants. VIA model has three axes, vulnerability, uncertainty, and support and assistance which determine the social suffering of the migrants that in turn influence their return. The intentions and return motives construct diverse return narratives, the various types and tropes of return discussed in section 2.2.

### 3.1 Theories of return migration

#### 3.1.1 Neo-classical and structural approaches

The neoclassical approach to return migration considers it as a return of failure. Migration here occurs when expected income is higher at the destination than at the origin, assuming the immigrants are risk neutral. It then promotes the idea that migrants have not been successful in their economic goals to get the expected income and, hence, their return. On the other hand, new Economies of Labour Migration (NELM) considers return migration as the initial plan, a calculated strategy to attain certain goals despite lower expected income at the destination than at the origin. The structural approach does not view the return as an individual decision. Rather, it takes into account the socio-economic factors at the place of origin and considers the source location as the decision-maker of migrations that are temporary in nature. The Social Network Theory, which expects the continuous contact of migrants with their kin back at home to influence the migration flow, conceptualizes the returnees equipped with tangible and intangible resources. The theory depicts returnees as “migrants who maintain strong linkages with their former places of settlement in other countries.” These “linkages reflect an experience of migration that may provide a significant adjunct to the returnees’ initiatives at home” (Cassarino, 2004).

#### 3.1.2 W-curve theory

The W-Curve theory postulates a reverse cultural shock model. Gullahorn and Gullahorn (1963) extended Lysgaard’s (1995) U-Curve theory^8^ of cultural shock describing the experiences of migrants (Norwegian Fullbright fellows in the US) settling in a new surrounding. The term cultural shock was first used by an anthropologist, Cora DuBois, in 1951 as a disorienting experience of an anthropologist’s encounter with a new culture. As return migration becomes a recurrent motif, the idea of reverse cultural shock begins to define the discrepancies felt at home after return. The W-curve theory combines two connected U-shapes to form a W curve, linking initial cultural shock with the reverse cultural shock. The returnee experiences an initial comfort on his or her return but gradually feels a cultural shock when the return expectations are not met at home. “As a consequence of the resocialization experience in the alien environment, a sojourner tends to acquire expectation patterns compatible with his new social system” (Gullahorn and Gullahorn, 1963). The migrant needs to start the readaptation process anew until settled, resulting in a W curve. The W-curve of acculturation and reacculturation experiences involves two variables of interaction and sentiment, the two interactions of two important concepts as discussed in the following point.

#### 3.1.3 Acculturation and cultural identity

Acculturation defines the post migration processes of the migrant. It “refers to the process of cultural and psychological change that results following meeting between two cultures” (Sam and Berry, 2010). Traditionally, acculturation occurs when different groups of individuals from their respective cultures are in continuous “first-hand contact”, causing effective change in the original cultural patterns. The basic difference between acculturation and migration lies in its anthropological significance. Migration is the physical relocation that can be plotted on maps whereas acculturation is the psychological adjustment. An individual conveys his or her original culture through emigration to a new culture. Acculturation hence becomes “the study of how people with one culture negotiate adjustment as they settle and adapt in a new culture” (Bornstein, 2017). The initial theorization of the 20th-century acculturation noted unidimensional and unidirectional models of change where the immigrants renounced their culture of origin for a new one. However, the changing migration dynamics with regular returns bring new negotiations for an immigrant’s navigation. The model, hence, becomes insufficient to highlight the return migration experience with complexities of cultural belonging and identities. The response to the limitations of acculturation studies can be seen in the concept of reacculturation as a consequence of “re-entry” (Arthur, 2003) which explains the cultural transition of returning migrants. “Reacculturation includes dealing with the difference between expectations and the reality of returning to one’s home country… a psychological process rather than a physical relocation to one’s home country” (Sonnenschein et al., 2021). It refers to the process of readjustment to one’s culture after acculturation to an alien culture, i.e., having lived in a new culture for an extended period.

Sussman (2010) in order to theorize the cultural identity about return migration proposes four distinct return migrant strategies, namely *subtractive*, *additive*, *affirmative* and *intercultural*. The subtractive approach tends to make interaction with other fellow returnees without any consideration of the previous host land. Return migrants adopt an additive strategy as an addition to the existing community at home by maintaining close ties with members of the left-behind host culture. “The experiences of subtractive and additive identity shifts are caused by obscured pre-immigration cultural identities which become salient after migration” (Kunuroglu et al., 2016). The affirmative approach, on the other hand, tends to strike a balance between both the previous host and current home culture by ignoring the discrepancies and differences of the two cultures. Return migrants, however, manage the shift between two transcultural belongings and cultural identities. This intercultural strategy, though, leads to a smooth return process, the identity shifts are characterized by stress upon return.

### 3.2 Return narratives: types and tropes

The narrative of return in its literary reflections is largely heterogeneous depending on the country’s immigration policies, conditions in the homeland, financial status, the standard of living and, most importantly, individual decisions. Hence, it becomes imperative to understand the multiple categories of return and how and why they occur. “Return has been defined and located in time and space, and how the returnee has been depicted” (Cassarino, 2004). Due to its complex nature, return migration is also conceptualized as return flow, reverse migration, reflux migration, homeward migration, U-turn migration, second-time migration, counterstream migration, repatriation, “ethnic return migration” (Tsuda, 2009), retro-migration and re-migration and cannot be defined in a set pattern. Bovenkerk (1974), one of the earliest definitions of return migration, describes it as people’s return after emigration for the first time to their country of origin. King and Katie (2022) defines return migration as the process when migrants return to their country of origin after spending a significant period abroad. According to Bartram et al. (2014), return migration occurs when migrants return to their homeland after the fulfilment of their original intentions or because of revised intentions. Erdal (2017) refers to it as the movement of migrants back to the source country after an absence of a minimum of one year. Akanle (2018) on the other hand categorizes a section of return migrants who on their return, drain the resources at home. The diverse and dynamic evolution of return migration over the years can be classified under several categories based on the nature and duration of return. The following table illustrates the major typologies of return migration theorized from 1974–2022. The wide timeline helps us to locate and understand the changing patterns of return with the increasing digitized infrastructures facilitating migration. Starting from Cerase’s (1974) fourfold typology to Bilgili’s (2022) transnational returns, return migration is classified based on the level of development of both the source and host countries, skill of the migrant, duration of stay, distance, motivation and circumstances of return. Following a bidirectional approach, transnationalism results in multiple forms of return, as shown in Table 3, Types of Return Migration. Bilgili (2022) discusses post-return as a process of reintegration with the homeland where the “reintegration may be hindered or facilitated by returnees’ sustained transnational engagements or belonging.” Post-return can be conceptualized as the lasting impact of transnational engagements abroad which influence the daily activities of migrants and continue to be a part of their belonging after they return. The detailed analysis of post-return as a significant return migration concept is discussed in the following section, where we trace the myriad nuances of homecoming.

**Table 3:** Types of return migration.

Category	Typology	Year	Types	Basis
I.	*Cerase’s fourfold typology*	1974	Return of failure, conservatism, innovation, and retirement.	Stage of development
II.	*Gmelch’s composite typology*	1980	Temporary or permanent return, remigration and circulation migration.	Duration and reasons
III.	*The return visit typology*	2001	Routine visits, ritual, care, root, rights, pre-return, economic and leisure visits.	The nature of visits
IV.	*Cassarino’s conceptual approach*	2004	Returnees with high and low level of preparedness or non-existent preparedness.	Preparedness and mobilization
V.	*Battistella’s fourfold typology*	2018	Return of achievement, setback, completion, and crisis.	Intend to migrate
VI.	*Transnational returns*	2022	Return visits, temporary relocation, long-term and ancestral return, imaginary return, and post-return.	Transnational belonging

### 3.3 The notion of return and homecoming

I think the very term “home” has two aspects of it, just as a concept. One – something to do with the normalized, the naturalized, the inevitable, the original. It’s there – the “thereness” of your existence, even more than the “hereness” of your existence. It is always there; this is my home. I understand this landscape. I know these people. I know the language, and so on… And the other, it seems to me, is the kind of Conradian idea that home is what you return to.

Return home, and the notion of “homecoming” (Vathi and Russell, 2017) is a common trope and sometimes a dominant theme in contemporary literature. Bhabha and Klaus (2017), in the above excerpt from his interview, talks about the notion of home, which emerges from the two intermingled ideas of diaspora and home as the “thereness of home” which always persists and the idea of returning to home. He goes on to say that “these two moments of temporality, these two narrative moments – coming out of the home and somehow allowing yourself to imagine, whether you can or you cannot, that you can go back: so, emergence and return are complicit with the concept of home.” Return migration or return home is seen as a reintegration with the homeland, closer to the roots and everything, it signifies. This idea of coming back to the place left behind forms the narrative of return and the trajectory of migration as experiences in the postcolonial era. Return, however, does not follow a set pattern and is heavily dependent on “distinct forms of narrativity, choices, judgments, which evaluate certain locations, which create a home around certain locations” (Bhabha and Klaus, 2017). The return reflects the reason for leaving, as discussed in section 2.1 of the study. As an increasingly recurring feature, return home defines a transnational affiliation that is characterized by a sense of belonging at both places. However, temporary return migration often results in a sense of placelessness, a feeling of neither being here in the homeland nor there in the host land, “a cultural shock, a trauma or a new displacement” (Stefansson, 2004). Juxtaposed against the idea of homelessness, Wood (2014) conceptualizes “homelooseness” ^9^, wherein the ties that bind a migrant figure to the homeland have been loosened on a temporary basis. If the connecting ties with the homeland are completely severed, then it would lead to a permanent migration without any possibility of return.

The aching desire for homecoming, even the conflict with home and the phantasmagorias home, hence becoming a central tenet of postcolonial literature. Migration is central to postcolonial literature “and to its analysis, both theoretically and concretely. It is also a kind of metaphor, a symbol that catches many of the shared understanding and assumptions which give postcolonial studies its parameters and shape” (Smith, 2004). Postcolonial migration is no longer a unidirectional path from the periphery to the center but transgresses several spaces, which do not create one single and stable home. A migrant’s perspective of the homeland and his or her identification with the home decides immigration and, more particularly, the return. The idea of a home anchored to a fixed location is necessitated by emotional and psychological connections. The paradigmatic shift of home from a physical motif to a psychological one is noted by Martin et al. (1986) when they refer to the state of being at home as familiar and safe in protected boundaries contrasted against the absence of home as an “illusion of coherence”. As an embodiment of traditions and culture, returning home becomes a journey to one’s own roots. In her essay, Spivak strongly dismisses the idea of tracing roots, saying, “If there’s one thing I distrust, in fact, more than distrust, despise and have contempt for, it is people looking for roots. Because anyone who can conceive of looking for roots should already, you know, be growing rutabaga (turnips)” (Spivak and Sara, 1990). Returning to connect to one’s roots, discussed as root visits, seems like a result of nostalgic yearning for the memories in the homeland. Hooks (1989) recalls her journey away from home as a necessity to move beyond the boundaries of the nation and the self. At the same time, she acknowledges the need to return to open new dimensions of perspectives, “I had to leave that space I called home to move beyond boundaries, yet I needed also to return there…Home is that place which enables and promotes varied and ever-changing perspectives, a place where one discovers new ways of seeing reality, frontiers of difference.” Returning home serves as a testimony of the mobility and fluidity of migrants, makes it easier to switch between their transnational belongings. These journeys back home form a fundamental part of the migration experience, a sacred pilgrimage^10^ symbolized by tokens of belonging and identity.

Human mobility has become easier and cheaper with the extended reach of digital migration infrastructures which enable a migrant’s presence at different locations simultaneously and leads to a “mobile lifestyle”^11^ resulting in the concept of place polygamy and multi-locationality, i.e., multimodal locations away from the locus of home. We will be looking at how DMI helps maintain ties with home and facilitates the return to the homeland in the following literary analysis section.

## 4 Methodology

The paper follows a close reading of the selected texts, visualization techniques, “tabulating word occurrences” (Horvat et al., 2015) and comparative analysis using the timeline and settings of each of the novels. Concurrence analysis using Python3 is a novel technique for textual analysis and concurrence mapping. The methodological design of the study involves a structured framework, systematic analysis of textual data, and extraction of meaningful insights through concurrence and co-occurrence analyses. The approach eases the interaction with large volumes of text. The methodology is an exploratory study that aims to unearth patterns and relationships which might not materialize through the conventional reading of texts. The quantitative tools are incorporated within the qualitative framework to provide the measurement and visualization of patterns in the textual data. In this way, the dual designs ensure the comprehensiveness and strictness of the argumentation in the study and presents a multidimensional analysis of the texts. The code is designed to take a single input from the user in a PDF or docx format, but for efficient operation and hassle-free toggling and analysis of different texts, the input functionality has been facilitated through a designated directory. The code was initially designed to take a single input from the user in a PDF or docx format, but for efficient operation and hassle-free toggling and analysis of different texts, the input functionality has been facilitated through a designated directory.

The code leverages the *fitz*, a high-performance library for data extraction, analysis, conversion, and text manipulation, and it enables seamless text content extraction from PDF documents. Through a series of specialized functions, the code systematically processes the uploaded file to identify keyword occurrences and pinpoint specific pages where these keywords are located. The keywords are entered manually through the user’s own understanding of the subject, on digital infrastructures and ICTs involved in migration. The code then executes a comprehensive analysis encompassing keyword counting, page-level keyword identification, and contextual snippet extraction for enhanced understanding. It further classifies the percentage existence of the keywords in the text into five major different classes like “Universal existence,” “Maximum match,” “Mostly match,” “Average match,” and “Poor match.” The code employs visualization techniques, such as map using QGIS and bar graphs generated using the *matplotlib* library, to provide intuitive representations of keyword distributions to enhance interpretability and facilitate rapid comprehension of key findings within the uploaded document. Results are saved to an Excel file, facilitating comprehensive documentation and reporting using the *openpyxl* module. The code’s modular architecture and well-defined functions improve clarity and ease of maintenance, enabling seamless modifications and expansions in the future. This aspect contributes significantly to text analysis, offering researchers a user-friendly instrument for extracting valuable insights from textual data. The computational methodology hence presents a structured framework for text data analysis, with an effective usage of Python to simplify the process and derive meaningful insights in context to our study of digital migration infrastructures. Coupling Python’s potential and capabilities for keyword extraction, frequency analysis, and data visualization with literary scholarship extends the breadth and depth of textual analysis to involve a whole novel instead of mere passages. Python’s effectiveness in this study is rooted in several factors (a)flexibility and customization as Python allows wide and ample customization, which permits the development of specialized functions that are tailored to the requirements of the study (b) efficiency in handling large volumes of text as the works analyzed in this paper range into several hundred pages and (c) integration with visualization tools to increase the understandability of the results. The methodology also involves preprocessing techniques to prepare the texts for concurrence analysis like cleaning the text and tokenization. It assures that the analysis is accurate and reliable by removing the content from any extraneous material that might affect analysis, be it headers, footers, or metadata.

The study also follows a purposeful sampling strategy that ensures the sample represents the larger corpus of interest. The selection criteria are relevant to the research questions, choice of keywords, and quality and integrity of the text. It involves identifying a range of texts from varied sources: articles from academic journals, reports, and other documents. The methodology for the selection of texts and keywords is based upon an extensive literature review of Indian English novels from of 2000–2023. We have conducted a comprehensive study on the digital intervention in the literature produced during this time. After closely looking at the various set of novels, the selection of novels takes into consideration the extent of digital infrastructure facilitating migration. The common thread between the three selected novels is that all of them are migrant narratives centered around the return experience of the characters. The protagonists of the novels have at some point immigrated to England or Canada, but still maintain their ties with the homeland through routine visits and return. The returnee experience in India hence becomes the base of the selection. We have then narrowed down the selection where digital infrastructures enable the migration process and affect migrant lives. The selection criteria hence is fourfold where Indian English novels within the (i) period of 2000–2023 as (ii) narratives of immigration, articulating the (iii) return experience specifically, with (iv) dominant digital intervention are shortlisted. The selection also is dependent on the availability of texts in a digital or PDF format for the concurrence analysis. There are certain texts that are suited for our study but their unavailability in the digital medium has restricted the scope which is also discussed in the limitations section. The choice of keywords determines the focus and extent of the research. It differs from each novel, after reading each of the texts closely. A novel published in the year 2000, *A New World* has negligible digital intervention. For that purpose, we have incorporated a few keywords based on non-digital forms of communication and physical infrastructures. These keywords like “letter,” “telephone,” “messages” in the following novels not only form a point of comparison but also traces the pattern of development from the physical to digital infrastructures in the process of migration. Some keywords also aim to decipher the dominant motif of the novel. Though the set of keywords are different from each other, based on the respective narrative structure, the primary focus lies on the digital and ICT keywords as represented in Figures 5A–H. The keyword selection methodology hence follows a preliminary reading of the texts, findings are later cross-referenced with the existing literature to ensure the comprehensiveness of the selected keywords. The final list varied between novels according to the individual focus, yet a suggestion of commonality held the broader analysis together.

## 5 Visualizing the migration landscape of India through selected texts

For immigrants who have fled to the US to escape, or to protest oppressive regimes in their homelands, immigration is loss of community of language, and of extended family. It is to give up on the dream of a better future in one’s home country. It is to cut oneself off from history and to condemn oneself to a world of ghosts and memories…It is to trust few but family and to feed family stories of horror and heroics in the abandoned country—(Mukherjee, 2011).

Authors of the Indian Diaspora have largely contributed to the discourse of postcolonial literature in its representation of the transnational Indian migration experience. Job, education and business opportunities in the developed nations abroad have been an alluring offer for Indian immigrants. The representation of Indians settling temporarily or permanently in American and European countries has become common in Indian English literature. “It is true that the migration of peoples is perhaps the definitive characteristic of the twentieth century. But, first, not all these shifts result in the same kinds of “diasporic” identities” (Loomba and Kaul, 1994). And though the Indian diaspora community is ubiquitous and largely dispersed across the world, every migrant experience is different from each other depending on the individual migrant idiosyncrasies.

**Figure 3:**
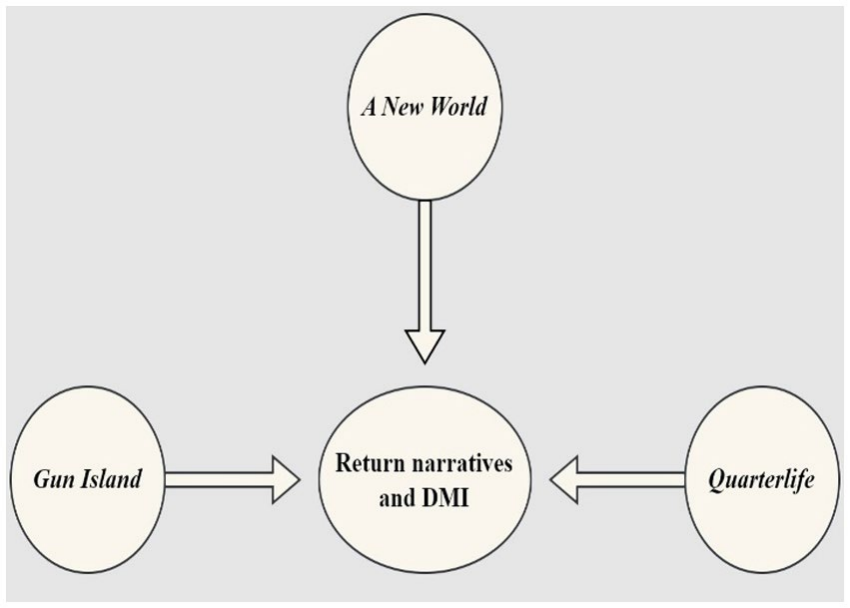
Selected texts.

The study conducted by Hron (2009) reveals that there are exceptions to the longing desire to return as the representations in novels about migrants’ conclusive return home are few. Despite the success and stable conditions at home, these migrants, prefer to settle in their new country. However, for those who return, their return home is often perceived as a triumphant success. To trace the patterns of return migration in India, we have selected a sample of three Indian English novels ranging from 2000 to 2023. The period of selection is closely related to the increasing use of ICTs and advanced digital infrastructures facilitating and connecting migrants. The visualization in Figure 3 depicts that DMI lies central to these literary narratives of return. We have sorted the selection based on the common threads in each of the texts, which make them a potential site of study for the analysis of Digital Migration Infrastructures (DMI). These novels are centered around returning migrants back to India, with return migrants as the protagonists of the narratives. Apart from the main character, these novels exhibit other characters as returnees or individuals and families directly connected to the returnees and how it impacts their lives. At the same time, these narratives have a limited or strong representation of DMI depending upon the period in which they are written or set. As novels set across a twenty-year span of time, the narratives offer a panoramic view of return migration and the evolution of the use of digital infrastructures in literature.

### 5.1 *A new world* (2000)

The first text in our analysis, Amit Chaudhaui’s fourth novel, *a new world*, centers around the protagonist’s migrant explorations in one fine summer season in Calcutta. The nuances of an Indian immigrant’s return find deft portrayal in the protagonist’s mundane explorations and ruminations. Jayojit, an Indian immigrant living in America as a college professor, visits home with his son, Vikram, during the summer break at school. The novel opens with Jayojit’s arrival in Calcutta, emphasizing his return home, “He had come back in April, the aftermath of the lawsuit and court proceedings in two countries still fresh, the voices echoing behind him. But he felt robust” (Chaudhuri, 2000: 1). As the series of events take place and the plot unfolds, we see that nothing really happens in the novel. The plot weaves around the daily Bengali Indian household, of family meals of “luchi” and “mach”^12^, morning newspapers, the hot and humid Calcutta summers, sleepy afternoons, evening table tennis, medical consultations, the nosy neighbors, Jayojit’s failed marriage, the mundanity of Admiral and his wife’s marriage, Vikram’s acculturation with his own culture and a returnee’s ruminations until he boards the flight back to California. “Nothing, after all, happens in them; pages are expended describing, in exquisite prose, the cursive curl of a letter or someone dozing off. Written seemingly out of life, these books are beautiful, intensely observed, yet static and inconsequential – more mood pieces than novels” (Mukherjee, 2015). However, Chaudhuri’s “plotless mediations” are reflections of larger understandings and perceptions of an individual’s migrant life and his or her transcultural belonging.

The author, from the very onset, sets the tone of a returnee protagonist who, throughout the novel moves back in time and space to his life in Claremont. The time difference between India and America struck him at the very moment of his arrival at 11 am, which he then calculated as 10 pm the previous night in America. The idea of time and the space he has left to come for his present visit becomes a part of his subconsciousness, a co-existing dream-like parallel world. He experiences a sense of dislocation as he tries to settle in at his own home in Calcutta, which he has hardly visited twice in the last ten years. “[H]is body seemed to be functioning to another time, or not properly to his one; but he had experienced this dislocation before and could ignore it. It was the previous night now in America; already America had become dream-like” (Chaudhuri, 25). His son, Vikram, also mentioned in the text by his pet name Bonny, is not bothered by the jet-lag. It is only his second visit to his hometown and thus his needs to reacculturate as a second-generation return migrant—“[H]e did not see, in the least troubled by jetlag, but seemed to have been remade and reshaped by this new climate, standing there, watching his grandmother” (Chaudhuri, 25). The novel hence presents two generations of migrants’ return to the homeland, one who was born and grew up here and the other, as a child born of Indian parents in America, who occasionally make return visits to his grandparents during his school holidays. For Jayojit, his return is more of a routine visit to maintain his ties with his birth country and his parents there. The first-generation migrants’ children become the next generation of return migrants. Bonny here forms the second-generation return migrant cohort—“They are the second generation (born overseas to an emigrant parent or parents)” (Conway and Robert, 2016). He makes sure that Bonny also gets to visit his place of origin in the form of a return visit, which can be understood as a part of root visits, discussed in section 2.2, where the journey between the two places shapes his identity.

The contrast in his parents’ conversing depicts the difference in native communication based on how the individual perceives the returnee. For Admiral, talking to his son in a few words and sentences in English strikes a note of formal initiation into the homeland from foreign shores. Jayojit’s mother uses the familiar and homely mother tongue to comfort and establish distant familial bonds. Chaudhuri’s use of the phrase “mother’s homely, slightly irritating Bengali” comes from the conventional idea of a NRI (non-resident Indian) returnee back to his homeland after years, only to find it difficult to adjust to the changes in language, climate and food habits. A similar strain is noted in Jayojit’s request for boiled water, which is suitable for drinking for his seven-year-old son. The assurance comes from his mother that Jayojit has had nothing but boiled water since he was a child. Jayojit is unaware of the practices at home that he has experienced since childhood, but at the same time, he is very particular about the habits carried from his life abroad as a return migrant. In recalling his life at Claremont, he draws a vivid depiction of a typical day spent at both places. The mornings were quiet in Claremont, a still and calm presence before the ringing of the alarm and springing back to life, contrasted against the mornings at his home in Calcutta, which are characterized by the noisy clatter of the kitchen, Statesman newspaper, the housemaid and a “dhobiwala”. Another important part of the morning routine is the elaborate breakfast of Bengali delicacies. Jayojit’s distaste for the daily oily and fried breads makes him conscious of nutritional value and diet control. This awareness comes from his food habits abroad which he carries back to India, and within two weeks of return, he decides to switch to the healthier to toast and tea, “In America he’d imbibed clear ideas, while having no idea that he had, of what to eat and what not to” (Chaudhuri, 52). Food becomes the most important part of the day for his mother, and she has a responsibility to feed her son and grandson, whom she believes are deprived of good food in America. To her, good food refers only to the Indian food that she has been preparing all her life. “She had never been abroad; it was an imaginary palace for her, territory that intersected with her life without actually touching it, and which had, for her, its own recognizable characters” (Chaudhuri, 46). It is with the return of Jayojit and Bonny that she gets a second-hand experience of the American lifestyle and gets a chance to appreciate and negate it at the same time. These little inclusions of the parents’ narrative in the text offer a larger commentary on the nature of transnational belongings and the families’ adaptation to it.

Jayojit turns out to be restless and bored within the first thirteen days of the two-month long visit. Feeling out of purpose, he just sorts to spend time reading newspapers. The description of his readings provides insights into the Indian notion of migration; migration as a better prospect is always perceived by developed first-world nations. “He read the papers twice, bored the first time, with the writing and with life in India, and in a more interested way the second time round; then he read an article about how well Indians were doing “abroad”; naturally, by abroad the reporter meant not so much Kuwait or Bangladesh but principally America” (Chaudhuri, 92). He disagrees with the reporter, condemns the Indian editorials, and prefers the American “vigilant candor” of *The Times* and the *New Republic*. Slowly, the narrative reflects upon how Jayojit, as a return migrant, develops a dislike and disdain for the things very Indian: the food, the reporting, the weather, the traffic, the commotion on the streets and the books by Indian authors. He not only remakes Indian books to be lightweight but also light in their significance. And when he ends up buying a book, the only satisfaction to him is that it is way cheaper than any book he would have to pay for in New York. The living standards in India and the expenses are far below those that he bears in America. Migration comes at a huge cost, involving both tangible and intangible resources. However, the shining prospect of life in New York still lures thousands of hopeful Indian migrants. “New York is attractive to every kind of Indian, from taxi drivers to dentists” (Chaudhuri, 122). The novel depicts the familiarity of a class of Indians with American pop culture and media. As Beatles and Lennon fans, Jayojit blended seamlessly into the new culture. Their acculturation to the new land was, hence, convenient without any major challenges. Jayojit’s return, rather, appears to be challenging in the readjustment to the Indian way of life. Every day of his return, he keeps discovering new peculiarities of the country, suggesting a lost touch with his homeland. “I keep forgetting how quickly it gets dark here,’ said Jayojit, as if it were yet another peculiarity he discovered” (Chaudhuri, 146). These arguments reflect his post-return condition, where the process of re-acculturation becomes challenging. Post return can be defined “as an incorporation for individuals who had already experienced difficulties fitting into the social and productive structure of their country of origin” (Parella and Petroff, 2019). In his re-insertion to the newly seeming environment of his homeland, Jayojit feels uncomfortable and rattles to be at home.

As a child, Jayojit has been travelling from one place to another, owing to Admiral’s shifting posts at work. His home continuously shifted from Bangalore to Delhi, Vizag and Cochin until he ended up in America. From having travelled since childhood, he comes from a privileged position; mobility between continents apart for him is as easy as buying an affordable “21000” ticket, a means to bridge the gap between his two lives of belonging. However, it is America that he relates to more, a place where he has finally found his home after years of migration from one city to another. Upon his return, his homeland seems to have become alien, and its way of life is astonishing. Despite being on continuous move, a sense of dislocation pervades within him. The author articulates it on his departure from Calcutta when Jayojit is onboard a connecting flight Claremont via Bangladesh, “But Jayojit had also experienced a slight feeling of dislocation when he’d realized that, although they’d left Calcutta at half-past seven, it was still seven-thirty in Bangladesh” (Chaudhuri, 232). Dislocation, according to Bhabha, transforms the liminality of the migrant experience into a translation of culture. The dislocation of culture pertains to a sort of cultural translation in a hybrid form that interacts and involves contemporary cultures that are otherwise in conflict. “Bhabha counters traditional sociological theories of migrancy that involve ideas of assimilation or acculturation: in his account, the migrant transforms the receiving culture, not vice versa. As a result, the dominant culture gets culturally translated by the migrant” (Young, 2017a,b).

Towards the end of the novel, Jayojit recites some lines from the Upanishads, which hehad read in English, “‘He moves, and he moves not,’ he remembered moodily, reciting to himself, with grim satisfaction, the lines describing the Spirit in the Upanishads. ‘He is near, and yet he is far.’” (Chaudhuri, 232). These lines are symbolic of his journey, his return visit to his homeland, and his return to the place he perceives as home. He is near his home but also very far from it at both locations. Home for the protagonist is, hence, not a single conclusive closed space. The novel finally concludes with Jayojit discovering a lump of “gur” (jaggery) inside his bag. It will be surprising for the American customs officers as this brown lump seems more like a clod of earth, the soil, and the mud of his motherland, which his mother carefully packed as a token of their return. This clod of earth is highly ironic as a symbol of a token from the homeland, which resonates with “the relics brought back from religious pilgrimages” (Baldassar, 2001).

### 5.2 *Gun Island* (2019)

Migration remains a connecting thread in Amitav Ghosh’s body of works. Human mobility becomes an appealing subject in most of his novels, ranging from his very first to his most recent novel. *Gun Island* (2019) as a significant novel in his career of fictional writing and an extension of the earlier novel, *The Hungry Tide* (Ghosh, 2006, 2021), represents climate-induced migration as a global concern. Set predominantly in the Sundarbans and moving forward in Los Angeles, California and Venice, the “novel portrays extensive out-migration of the region’s isolated agricultural communities along traditional migrant corridors into Northern Africa and Southern Europe” (Wilton, 2021). The central premise of the novel is set on migration and refugees and stories that emerge from these migrations. And it is the story of “bundook”, which itself travelled to Bengal far from the shores of Venice, which remains an enigmatic conundrum until unravelled towards the end of the novel. “Bondook” meaning gun in English becomes the title as Gun Island wherein the narrator attempts to demystify the legend of “bonduk dwip” and the shrine of “manasa devi” in Sundarbans. Ghosh here represents the literary landscape of this 40,000 sq. km island, highlighting the pressing concerns of climate change, migration, language, folklore, mobility, and borders. He sketches the tidal country in his fiction as a site where the history of colonization failed to build a settlement owing to the wild nature of the land and its predators, and the continuing exploitation leads to hundreds of refugees into the mainland and illegally across international borders. The issues of refugee movements, migration, the disruptive and constitutive flow of global colonial and neocolonial capital, the etching left by these on communities and lands, the dynamics of resistances and appropriations-in other words, nothing less than the cultural and historical environments of colonialism and postcolonialism – have long been at the heart of Ghosh’s work” (Mukherjee, 2010).

The narrator of *Gun Island*, Dinanath Datta, returns from Brooklyn to Kolkata as more of a routine visit to his homeland. In his years spent as an immigrant in America, his hometown becomes more of a nurturing shelter, as he states in the opening lines of the novel, “Kolkata was also sometimes a refuge, not only from the bitter cold of a Brooklyn winter but from the solitude of a personal life that had become increasingly desolate over time, even as my professional fortunes prospered” (Ghosh, 2019: 3). A rare book dealer by profession, Deen meets Kanai in Delhi, leading him to Nilima Bose who wants to untangle the knots of this legend whose history can be traced back to the seventeenth century. As he sets forth on his journey to the Sundarbans, he gets a glimpse into the hardships of the livelihood there, the reality of human trafficking and the illegal migration of the natives. “[T]he exodus of the young was accelerating every year: boys and girls were borrowing and stealing to pay agents to find them work elsewhere. Some were slipping over the border into Bangladesh, to join labour gangs headed for the Gulf.” And on failure they would again pay extra to the “traffickers to smuggle them to Malaysia or Indonesia, on boats” (Ghosh, 49). As an American returnee, it becomes hard for him to comprehend the crisis of the land and adjust to the wilderness of nature. It is difficult for him to navigate through the muddy soil and he eventually tip over the deep mud. His return to India over the years has been one of privilege in passport counters and visa lines, business class tickets and air-conditioned rooms. The idea of climate refugees is a far-fetched idea for him, where the migrants escape the barbed wires and waterways illegally, often with fake identities. His belief in passports is more of an assertion of his identity, following the norms of being a legal citizen of the country. “I did indeed believe in passports, visas, permits, green cards…For me these were not just pieces of paper or plastic; they possessed a certain kind of sacredness that attached also to the institutions that issued them” (Ghosh, 59). As an immigrant and a frequent returnee, Deen has normalized the institutionalization of migration and its entailing procedure with a veneration for the system. Since Deen makes routine visits back to India, it becomes more of a habit to reverse his Indian and American identities, “Experience had taught me that to travel between Calcutta and Brooklyn was to switch between two states of mind, each of which came with its own cache of memory” (Ghosh, 103). Ghosh here reflects on how the idea of a seamless shift between two selves while retaining a distinct memory and identity of each one evolves as a distinct return migrant feature. While the narrator acknowledges that the memories of India seem to recede at his Brooklyn apartment, but his visit to the Gun Merchant’s shrine occupies his mind completely as he finds himself in helpless recollections of that day, so much so that he must seek medical help. “For several weeks after my return I found it hard to focus on my work” (Ghosh, 103). It is the unresolved mystery associated with the place that carried itself all the way to New York, and it is the same inquisition that brings him to Venice.

Ghosh extends the characters of *The Hungry Tide* in this novel, and we can see Fokir and Moyna’s five-year-old son has grown up here. Tutul grows up as Tipu in *Gun Island,* and it is in his encounter with Deen that the author highlights the growing business of the people moving industry. Tipu takes up the profession of an agent offering essential services to aspiring migrants, helping his clients with the logistics from one location to the other and offering appealing story stories of “politics, religion and sex” to ensure their asylum protection. Ghosh employs the “butchery metaphor”^13^ in the novel which “not only connotes the dehumanization of the migrants but foreshadows their transition from victims of the human trafficking industry into the products of an activity even more macabre” (Wilton, 2021).

The story of the gun merchant is revealed to be an apocryphal record of a real journey made by the merchant to Venice. In the attempt to decode his journey, Deen and his friend Cinta trace the route from Goa in India to Maldives to Egypt, Turkey and then finally to Venice. Ghosh’s well-researched narrative also exposes that the passages of illegal migration from Bangladesh and India to Europe also follow almost a similar route. The narrative unfolds to reveal that Rafi and Tipu have taken the perilous journey through the migrant corridors of the Middle East and Africa in attempts to reach Venice, only to provide cheap labour and pay back huge amounts of loans to the agents. Rafi follows the route from Sundarban to Dhaka to West Bengal to Pakistan to Iran to Turkey, ending up in Venice. Tipu gets stuck midway through Egypt due to a shortage of money to pay the agents. The author depicts Venice as a major European hub of immigrants where hundreds of Bengali-speaking migrants serve as cheap labor in the city. Most of the immigrants here are climate refugees who have suffered in their native places. Lubna, an immigrant in Venice for the past twenty years, recalls the devastating cyclone “a fearsome tufaan” that made her migrate across borders. The depiction of the city in the novel is through the eyes of an immigrant, represented in Deen where he is trying to seek the familiar through language. “Crossing paths with Bangla speakers in faraway places had always been for me a matter of pleasurable surprise…it would help me find a context for myself in this unluckiest of cities” (Ghosh, 163). As an immigrant making his living in the USA, Deen finds comfort and solidarity with people and places that share his mother tongue, Bengali, but remains ignorant about the adverse migrant conditions.

The narrative set in Venice largely reflects on the unrestricted flow of immigrants through illegal ways and the European refugee crisis. Ghosh reflects on the crisis mainly through the arrival of the Blue Boat of refugees navigating from the eastern Mediterranean to arrive on the shores of Italy. The “boatload of refugees” started from Egypt with several ethnicities, “Eritreans, Egyptians, Ethiopians, Sudanese, and maybe some Bengalese as well”. As this was the “first refugee boat to head towards Italy in a long time” (Ghosh, 173), the minister has determined to stop it. The author here portrays a fictional representation of the government policies and campaigns of anti-immigration and anti-refugee approaches. In the novel, migration finds a kaleidoscopic depiction in its manifold forms. Even in the novel’s concluding chapters, Ghosh introduces new characters as a scathing commentary on the migrant dream of a utopia. In the hope of studying at a European university, Palash invests all his savings for a student visa and a plane ticket and lands in Italy from Dhaka. A dropout from the University of Padova, his work permits are continuously being rejected, and with no possibility of return, Palash has been living in limbo for the last four years. The failure to live up to his dream comes at the cost of his return as the doors of his return back home have been shut in, not keeping up with the expectations of his family,

“My family still does not know that I dropped out of university and am now scraping by on the streets…If I tell them the truth now I would have to admit that I had been lying all along…to acknowledge that in chasing a dream I destroyed my life” (Ghosh, 267).

In the climax of the novel, the characters come together to rescue Tipu from the Blue Boat when a catastrophic tornado hits the rescue ship, Lucania. A harrowing sense of the migrant predicament excruciates the narrator. Young migrants like Rafi, Tipu and Palash set on their journeys to seek a better livelihood but are subsequently reduced as outcasts serving cheap labour to the white Europeans. Their status is almost similar to the imperial slaves, and chained laborers make their return all the way more difficult. “It was the desires and appetites of the metropolis that moved people between continents…In this dispensation, slaves and coolies were producers, not consumers; they could never aspire to the desires of their masters” (Ghosh, 279). Ghosh ends without offering a conclusive direction to the novel and the decisive future of each of the characters. Nonetheless, *Gun Island* ends with a strain of optimism in the spectacle of bioluminescence and in the rescue of the boat refugees by the government representative, Admiral Vigonovo. The calm sea and clearness of the sky reflect on the shining ray of hope for the boat people and thousands of other immigrants who cannot return to their homeland.

*Gun Island* portrays several return migrants engaged in a chain migration^14^. Deen, Piya, and Cinta all migrate across oceans to come back to their designated spaces. Since our subject of study here is return migration, the novel can be interpreted as narratives of returning migrants, whether it is the folktale of the gun merchant, the narrator-protagonist or the narratives of Cinta, Priya and Nilima. Ghosh here depicts the idea of migration in a digital age where it is no longer restricted to a one way process and where the access to homeland is facilitated through a nostalgic reverie, occasional letters or a diasporic visit^15^ once in a few years. Technology has intervened in migration to disrupt the conventional yearning for home by dissolving the distinct boundaries of time and space through the internet. The author here explores the technological exploits involved in migration and its infrastructure, as he clearly comments in one of his interviews on *Gun Island*,

“[I]n this book, I’m also trying to explore the uncanny aspects of this technology, such as the ways in which the Internet can also direct hate and give expression to the really malign forces of human society. And it can direct it with this incredible power. All of that interests me.”^16^

The novel can hence be interpreted as a commentary on the interplay of migration and the digital medium in Ghosh’s oeuvre of literature.

### 5.3 *Quarterlife: a nove*l (2023)

The most recent novel, in our analysis, *Quarterlife* offers a kaleidoscopic array of return migrants through its intricately woven narrative structure, which follows the life of three parallel characters. Devika Rege, in her debut novel, addresses the question of the identity of abroad-returned Indian immigrants and illuminates the notion of reverse migration from the West to the East, set against the backdrop of 2014 post-election rising nationalism in the country. The novel opens two months prior to Naren’s return and Amanda’s arrival, along with the author’s underpinning on each of the characters. Naren Agashe returns to his country after quitting his job in the US and turning down job offers from two other Manhattan firms. “After a decade in the States, Naren Agashe has booked a one-way ticket to India” (Rege, 2023: 10). For he believes that India, as a developing country, is teeming with a huge lot of economic prospects and that it now seems the right time to return with the rise of “conservative forces”^17^. Meanwhile, Rohit Agashe has yet to find his call. As an aspiring filmmaker, he aims for an “epic life” that is relevant and authentic. Amanda’s entry into the narrative is in the context of her arrival to India through her India Impact Fellowship when she requests her college classmate, Naren, to arrange her visit to India, where she will volunteer and work for NGOs in Mumbai. Rege and Sen (2024), in her interview, states that each of the characters offers an understanding of the migration and political scenario of the country, reflecting on a worldview of contemporary times. “Rohit, Amanda and Naren also offered a useful triumvirate of perspectives in the form of the insider, the outsider, and the insider-outsider.” The ideas of insider and outsider, returnee and foreigner, and the Western gaze and othering are pertinent discussions throughout the text.

Naren’s eight years stay in America was one of void without any feeling of triumph or relief that otherwise accompanies an Indian immigrant. And now, even with new job offers and a green card, Naren finds no purpose in staying back. “The job offer changes nothing; his existence in America is like bread gone stale, it elicits neither pleasure nor disgust, only a desire to toss it without much thought into a bin” (Rege, 7). His optimistic perspective that the Indian economy will be no less than that of the US pushes him back to his homeland. As discussed in the typology section 2.2, Naren’s return can be interpreted as the return of innovation^18^. He believes in the future of his homeland and moved by the newly elected prime minister’s speech, an appeal to every Indian to do best for their motherland, he terms his own return as something forward, “back is the wrong word, the word is forward…the future is not in the West, it is in the East” (Rege, 20). On arriving at the newly made Agashe home, he realizes that caught up in the American fetish of a self-made man, he has not considered the progress of his own family. The author’s commentary on this reflects that an immigrant unknowingly returns with an aura of self-achievement and triumph overseas, which overshadows the privileges at home. This idea lies in stark contrast to American migrants in India, as seen in Amanda. She is rather surprised that it is the first time that Naren has visited his new home since returning. Upon his return, Naren tries hard to maintain the demeanor of a global citizen by engaging in conversations that are otherwise unlikely of him, like gastronomy and art. His styling in a chambray shirt, chinos and leather loafers rather reflects his insecurity about his life back in India. Not ostentatious by nature, his effort in dressing up suggests “the shift in register when Naren tries to balance the lilt he uses in America with the singsong accent of home” (Rege, 32). Though he is vocal about Indian development, he still carries the American touch along with himself, whether it’s his lifestyle or his vision at work. Naren began his career in New York for all the flashy and grand things like a fancy car and condo, impressive portfolio and all the latest gadgets and fine suits. He talks about the pressure of maintaining the very Indianness in harmony with the Western identity. Unlike the various forms of return visits where the returnee is expected to bear a strong foreign influence as he again flies back, permanent return is also tinged with similar impacts. It is more of his rootlessness that is reflected in his attempt to strike a balance between both his lives, where the American self is always more biased than the Indian. This comes as an embarrassment for Rohit, to whom Naren signifies “the last of the brain-drain generation that truly wanted to be American” (Rege, 65). If we look into the VIA model, Naren’s return is “pushed by the vulnerability” of his “situation in the destination country” (Parella and Petroff, 2019). He becomes a victim of the social suffering in respect to the discrimination faced at his workplace, of being a brown Indian working successfully in a place surrounded by whites.

We can trace Naren’s journey from an aspiring migrant to a hopeful returnee. He had transgressed the borders of his “janmabhoomi” to shape his “karmabhoomi”^19^ continents apart. However, in the latter stage his migrant journey, his ideologies shift towards his “janma bhoomi” and on his return, India becomes the land of both his birth and work. Rohit evidently recalls Naren’s enthusiasm in starting his career in America, working ninety hours a week and securing the rank of top analyst. However, the glam of American work culture soon fades and becomes stained with racial discrimination, he soon discerns that he will always be treated as a second-class citizen, an immigrant with a brown skin color “[I]t seemed like both success and failure would confirm what he could not bear to admit: that in America, he was expected to come a close second, but not to win. His ambition, which had felt pure until that point, became corrupted” (Rege, 77). Towards the very end of the novel, he acknowledges that he has lost all faith in the American dream, and the need for new values is necessitated by his return with a new array of dreams and good days. “When I caught the flight to India, I was acting on instinct. I thought it might help to be rooted in my culture, or closer to family, or that working for my country would take the focus off myself” (Rege, 371).

Rege has sketched the character of Rohit in contrast to Naren, which offers further insights into the individual perspective to return to the notion of a new India. She puts it clear that Rohit has “no claimable talents- expresses itself in this excess energy, this sense that he is at the cusp of an era and wants to be at the center when it all comes together, to be at the center and to be young” (Rege, 17). He holds high opinions about his elite circle of two thousand graduates from Mumbai’s top colleges who represent the voice of the generation. However, he begins to question the same social niche with the recent win at the elections that propagates an exclusively Hindu nation with extremist ideologies, “All were active online, retweeted, even trolled… but since their collective shock at the Bharat Party’s massive victory, his suspicion has been confirmed: what he once thought of as a generation is really a clique” (Rege, 32–33). The mixed responses to the party in power are reflected in Naren’s and Rohit’s reactions to it. To Naren, it is a life-changing event, so much so that he has returned from his well-settled life in America to start anew to live to the expectations of a new India. Rohit, on the other hand, does not recognize the concrete reality of borders and the ideas surrounding them, like passports, homes and families. These are rather evasive ideas to him juxtaposed against the reality of urban Bombay life of colonial buildings and American brands.

The trope of return is also present in Ifra’s journey to make a place for herself. As animmigrant student pursuing art history and development studies in London, she comes back to her home in India, where she represents the minority community. Her decision to return and work in India is to pull back home to seek a sense of belonging. However, Ifra’s fervent homing desire^20^ is disillusioned by the newly elected Bharat Party and thereby questions her return. “The West never felt like home, but since the election, home does not feel like home either” (Rege, 123). It is the author’s articulation of the stressful socio-political conditions directed majorly at the minority population of the country. Now that she has a job offer in Dubai, she decides to leave India again for better prospects, and what haunts her is the fate of people from her community who have no option but to continue living here, “what about the people who cannot leave?” (Rege, 175).

“You’re all so young, barely past your quarterlife! You have all this time and energy, but unlike every previous generation at your age, you have nothing and no one to be responsible for, so you go chasing utopias” (Rege, 216). The novel’s characters are rushing to strive for success, and in this run for a better life, each one makes a journey. Sometimes, the journey is inward within the individual, and sometimes, it is outward across borders. Amanda’s journey in the novel is one of self-exploration and discovering the harsh truths of life until she is molested in the crowd of a Hindu religious gathering. She senses a deep connection to India even before her arrival, which might be traced back to her British grandfather or her aunt’s visit to the country as a haven under Lord Krishna. Amanda’s visit can be perceived ambiguously as the author depicts in the text. In her relationship with Rohit, she wants to find her new self, and it is through her work that she seeks an independent identity. “Like the children, he is extracting a new Amanda from her, the one she has travelled to this end of the world to meet” (Rege, 97). The postcolonial gaze interprets Amanda’s visit as an exotic adventure to the East, but at the same time, her dedication to work is serious. Dressed in Indian attire of kurta and scarf, backpack with camera and toilet roll, she is set to begin her work in the slum areas of Deonar, the country’s largest dumping ground in Mumbai. The objects mentioned in her backpack, with a special emphasis on the toilet roll, suggest her acculturation in India as an American, where she considers it to be a necessary object to travel with. Adjusting to the sweltering heat of the summer months has been a common concern for immigrants or returnees to India. As depicted in section 4.1 of the textual analysis, Jayojit’s complaint about the hot weather is recurrent throughout the narrative of *a new world*. In *Quarterlife*, it is not the returnee, Naren, who is seen complaining; rather, the author reflects on Amanda’s adjustment to the Indian climate. “Summer in the tropics is like winter in the West, weeks of extreme heat as harsh as extreme cold, her only recourse being to cultivate a mind for it…it was what she saw in every face: the face of summer, resolute against the burn” (Rege, 91). At work, she is assigned to create a “photographic archive” of an “unmediated confrontation” with the living conditions of the slum as a part of Ashray’s media outreach. Her Indian migration experience is fraught with challenges both at work and her relationship with Rohit. During this visit, she suffers a personal loss, the death of her grandmother, Nana, and returns home for the last rites, which leads to a ritual return visit, as discussed in section 2.2. Towards the end of the novel, the gaze of an outsider acts detrimental. Her presence at the country’s largest “Ganpati visarjan” as a foreigner makes her an easy target for the molesters. While Rohit is engaged in the dancing crowd, the mob takes the opportunity to attack and molest Amanda, an outsider, a white woman, who might be a threat to their religious orthodoxy “She scans the mob for a saffron headband or a drumming girl, but the faces closing in are strange and grotesque…Then the world is a sharp crack and pain, pain…The final sound is that of drums, swearing and drunken laughter” (Rege, 343). And while the city is praying for Lord Ganesha’s return the next year, Amanda’s skull is cracked open, never to return back to India. After this incident, she leaves India with scars, trauma, a sense of racial melancholy and a hope to recover from it. Her return back to New England is one of anguish and agony, suffering cast on her for being an outsider. Migration is commonly associated with mourning and melancholia over the loss of individual culture and heritage. “These losses combine with the melancholic effect of becoming minority subjects whose cultural and material exclusion is used to preserve an image of normal U.S. citizenship and subjectivity as implicitly white” (Chu, 2019). The notion of racial melancholy, also discussed by Cheng^21^, examines the material, cultural and political conditions of exclusions of white from the native experience.

**Figure 4:**
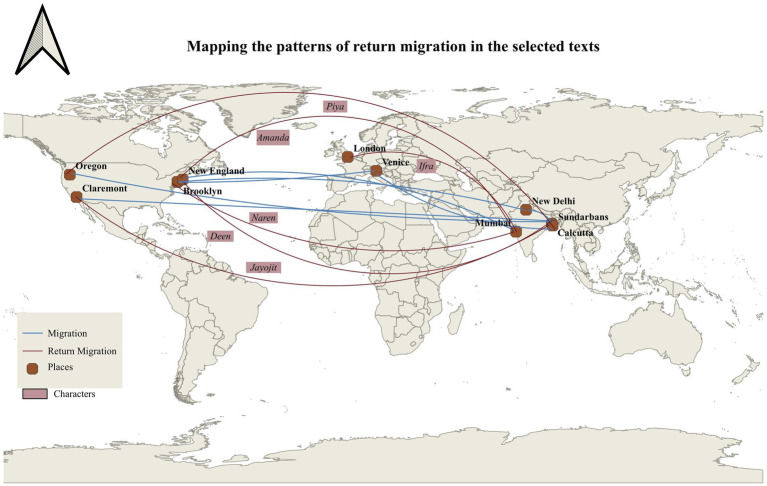
Mapping the patterns of return migration.

Return depicted manifold forms in these narratives are closely knit to the digital migration infrastructures, which we will discuss in the following section by studying the results of the concurrent analysis. The map (Figure 4) traces the patterns of migration of the multiple characters of the three novels in discussion. The protagonists of the three novels migrate and return, Jayojit from Claremont to Calcutta in *a new world*, Deen from Brooklyn to New Delhi and then to Calcutta in *Gun Island* and Naren in *Quarterlife* from Manhattan to Mumbai, never to leave again. Other important characters like Tipu and Rafi underpass the migrant eastern Mediterranean migrant corridors to reach Venice illegally as an escape from the wilderness of the Sundarbans. In *Quarterlife*, we can trace Ifra’s return to Mumbai after getting a degree in London. It gives a clear picture of the frequent places of migration from the metropolitan cities of India to the USA and parts of Europe, creating a circular movement.

## 6 Results and analysis

### 6.1 The early novels of 2000s

**Figure 5:**
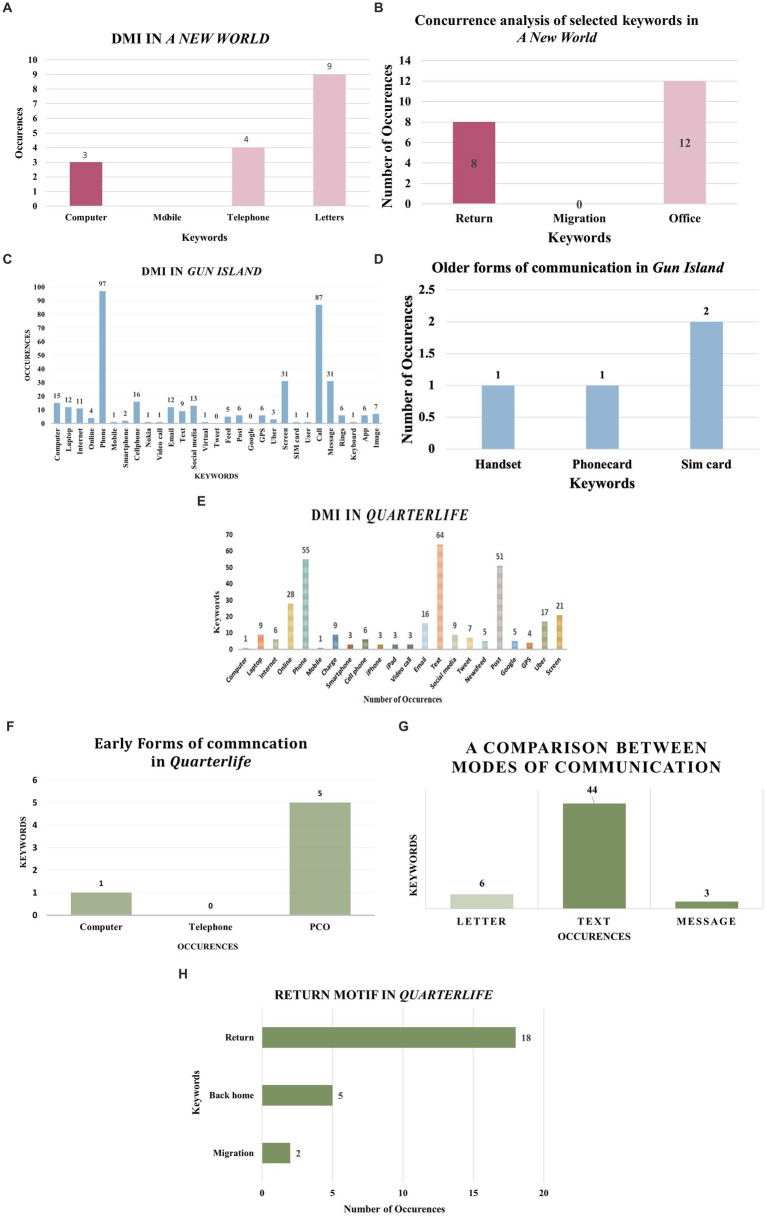
Concurrence analysis results. **(A)** Result 1. **(B)** Result 2. **(C)** Result 3. **(D)** Result 4. **(E)** Result 5. **(F)** Result 6. **(G)** Result 7. **(H)** Result 8.

In the concurrence analysis, we can see how a novel written in the year 2000 has negligible or limited engagement with digital infrastructure or ICT technologies. As seen in the graph, Figure 5A, the mention of the internet within the narrative has a poor match as it does not occur even once. In the visualization of the results, the word “computer” occurs thrice within the novel. The direct use of the internet is negligible, and the use of mobile phones can be seen only twice. Since the use of ICT is limited, non-digital platforms take center stage as writing letters is preferable and become a dominant way of communication rather than emails and instant messaging. “Three months after their wedding, Amla had begun to write to her mother-in- law from Arlington…They were determinedly chatty but formal, and full of questions, perhaps to camouflage some sense of inadequacy” (Chaudhuri, 2000: 171). The medium of letter writing was the only way of communication, other than telephone, before the active engagement of digital infrastructures in almost every household. For her mother-in-law, though not good at letter writing, it is more of a fleeting joy to interact and gain a distant experience of their life in America, “To Mrs. Chatterjee they had given a fleeting pleasure, and also the obligation of having to reply in simple, serious sentences that were, however, laboriously composed; for she was a poor letter-writer” (Chaudhuri, 171). The use of the telephone in the same way is the only device to talk across the space of time and place, as wireless mobile communication has no mention in the narrative. The author makes the two characters, Amala and the Admiral, use the telephone as a means of leisure, talking and passing valuable information. Admiral makes calls to his son hundreds of miles away to enquire about the legal proceedings for Jayojit and Amala’s divorce. Here, the author highlights the limitations of using a landline, “Another deliberate pause; because if you interrupted the speaker the words canceled each other out. You had to be sure the other person had finished. Sometimes there was an echo” (Chaudhuri, 107). He further states that Amala “began phoning her parents twice a week, speaking loudly over the mouthpiece as if she were talking to people in the next room” (Chaudhuri, 173). We see Amala using both communication methods, which were readily available at that time. The employment of letters and the telephone also reflects her understanding of communication with different people, as she adopts different modes for her own mother and her mother-in-law. Long and regular telephonic conversations suggest a close-knit relationship and proximity despite the distance, whereas writing letters thick with questions just as a formality makes both the receiver and the sender aware of the distance (physical and emotional) between them. Further, when Jayojit wants to reconfirm his ticket before his departure from Calcutta, he cannot do it on a digital device like a smartphone or tablet in a few seconds. Rather, he had to travel all the way to the airline’s office of Bangladesh Biman, “The Biman office was crowded: the queues disintegrated in a mixture of high spirits and panic.” The chaos and commotion of the airline offices and travel agencies are sorted by the intervention of DMI. Figure 5B depicts the occurrence of the office for booking tickets as a physical infrastructure of migration in parallel to the motif of return in the novel. In the later novels, we will see how the physical spaces of migration infrastructures are gradually becoming obsolete.

The intervention of Digital Migration Infrastructure is almost absent in the novel. As the earliest novel in our selection of texts, we see that there is minimum usage of digital devices compared to the other texts in our analysis. *Internet World Statistics* reveal that around 5.8% of the total world population had access to the internet in the year 2000, which has grown up to 69% in 2022^22^. We can hence conclude that the lack of availability of ICTs to the masses is also represented in the text as a novel of its own time.

### 6.2 Digital passages in illegal migration

“In *Gun Island*, voices and visions from the other world seem to compliment text messages from mobile phones and computers to communicate to the characters in uncanny ways that defy reason” (Kaur, 2020).

The human in the novel interacts with the technology in the digital space to migrate spaces across borders. It is of great interest to the author to explore the ways in which the digital aspect of human life affects and paves the way for migration (Gill and Ghosh, 2019), which is directly reflected in *Gun Island*. As “the movements of men are the object of heavier and heavier limitations” (Balibar, 2004), the digital migration infrastructure creates digital passageways offering access to distant lands in the hope of a better life. The concept of passages can be traced back to Benjamin’s (2002) the arcade project, wherein he debunks the symbolic connotations of technological developments. Arcades, forming a pedestrian passageway by connecting two streets, were the most significant architectural innovations of the nineteenth century. This sheltered roof housed many small cafes, restaurants and shops, containing a “dream and wish image of the collective^23^”. This dream and wish image also encapsulate the digital passageways, creating a digital platform to transpose users to their dreamland. Much like the French arcades, these passageways lead to the development of “negative experiences of exclusion and positive experiences of agency and empowerment.” “As digitized passageways, Internet applications offer advertisers a chance to market their products and monitor consumer preferences” (Leurs, 2015), which heavily influence their decision to migrate as depicted in the novel in discussion.

The concurrence analysis of *Gun Island* using DMI keywords from the text reveals the role of digital passageways in shaping routes and destinations of the migrants. The bar graph depicts the frequency of the keyword occurrences, highlighting the increased DMI intervention in the novel. In analyzing the results of Figure 5C, we can understand that the direct use of Twitter and Google are absent, but the use of social media platforms and internet surfing forms an integral part of the narrative of migration. DMI in the novel hence offers to its users like Tipu, Rafi and several others like them, “an enjoyable miniature world… a chance to enjoy themselves, observe other people” (Leurs, 2015). Tipu’s fluency in the digital medium is due to his increased interaction with phones and other devices. To provide for a better life, Piya offered Tipu all the technological advancements in the form of the latest laptops, tablets, and smartphones. However, this exposure to ICT in a rural area like Sundarbans serves to act otherwise. Tipu uses the digital infrastructure to promote the flourishing people-moving industry, linking the ties between migration and the digital. He works as an agent to sell stories to migrants seeking asylum, point-to-point service to clients about stories of politics, gender identity, religion, and sexual orientation. Here, it becomes important to note the role of social media as DMI, where without any advertisement or propaganda, the stories on the feed easily influence the users.

“It’s all word of mouth, going out on social media. A guy might see something that a a previous client has posted and then decide that he wants to take the same route. Or else he and his friends will just get curious. They’ll come and talk, and if they want to go ahead, and can raise the money, I connect them to dalals” (Ghosh, 63).

The “dalals” as Ghosh uses refers to an agent who bridges the link between the point of contact and the rest of infrastructure. Tipu is one such Dalal, connecting prospective migrants from one person to another through the phone. The payments and routes are communicated through the phone itself, “From there on, the phone becomes their life, their journey” (Ghosh, 62). Another role of DMI lies in maintaining contact with relations back at home. Tipu connects to her mother, Moyna, through smartphones and social media accounts. The international phone calls and messages, in turn, reveal his location, which helps Deen and Piya to trace his refugee journey without any proper means, resources and paperwork.

In his visit to the Manasa Devi shrine into the deep muds of the forest, Deen regrets the absence of his phone to record and digitally document the glory of a lesser-known 17th century Bishnupuri architecture and several symbols carved out on the red clay panels of the temple. As an immigrant residing in America and visiting the elite corners of India only on his return, Deen is oblivious to the expanse and reach of technology to the farthest remote corners of the delta island of Sundarbans. “It shamed me to think that I had assumed that Rafi would be unacquainted with the cellphones simply because he was a Sundarbans fisherman” (Ghosh, 85). Digital infrastructures have impacted human lives even in the remote corners of the mangrove island, even before the Internet became an imperative necessity. Going back to the cellular days, we can trace the influence of technology in determining migration in Palash’s narrative, where his return seems to be impossible. “[O]ur first cellphones were Nokias, made in Finland, so we always had a soft spot for that country…we all wanted to go to Finland, it was our fantasy, our dream” (Ghosh, 206). In contrast to the trending and latest technologies of smartphones, Ghosh also shares a tiny glimpse of the traditional phone booths and public phones in the city of Venice. The occurrence of handset and phonecard amidst the DMI keywords, enlivens the object of the recent past into the text, as seen Figure 5D.

As the dominant DMI, the internet has changed the idea of migration in contemporary times.

“The Internet is the migrants’ magic carpet: it’s their conveyor belt. It does not matter whether they are travelling by plane or bus or boat: it’s the Internet that moves the wet are…You do not need a mainframe to get on the Net- a phone is enough, and everyone’s got one now. And it does not matter if you are illiterate: you can call up anything you like just by talking to your phone—your virtual assistant will do the rest. You’d be amazed how good people get at it, and how quickly. That’s how the journey starts, not by buying a ticket or getting a passport. It starts with a phone and voice recognition technology” (61).

The short excerpt from the text here highlights the constantly emerging and growing intervention of digital migration infrastructure in determining migration paths. The access to DMI is not restricted to poverty, digital literacy or literacy at all. A phone, rather an affordable object to the masses, opens up new horizons of scope and opportunities for mobility across borders, legally or illegally, through migrant corridors. As seen in the passage above, Ghosh comments on the interdependence and interconnectedness of every technology, medium, and platform within the DMI. Individuals derive this need to migrate in an idea of a better life sourced from their smartphones and social media engagements. The visual lure of seeing the experiences of people they are related to creates an urge within them to get a first-hand experience themselves, hence getting entwined in a trap of something better across borders.

### 6.3 The recent DMI intervention

The intervention of DMI has significantly increased in migration literature, and specifically in the genre of return writing over the years. In both the close reading and computational analysis of the novel *Quarterlife*, we can locate a plethora of ICTs and DMI. The novel, set in 2014 and written in 2023, depicts the nuances of immigration, return, nationalist and communal feelings in a complete digital niche. The results of the computational analysis reveal the presence of a well-constructed digital migration infrastructure facilitating both the immigrants and the returnee.

The graph, Figure 5E, visualizes the occurrences of the selected keywords in the given text. A distant study of the results reveals the multitude of ICTs present within the novel. The older forms of digital infrastructures like computers and mobile occur only once and can be seen replaced by the more portable and advanced laptops, tablets or iPad and smartphones. Smartphone in the text occurs 64 times, “The smartphone and social media are almost universal communication tools that act as a lifeline to help maintain pre-existing family bonds across physical geographical borders” (Leurs and Patterson, 2020). In the novel, they frame the migrant and returnee experience. The word “phone” or cellphone is used throughout the narrative as a reference to smartphones, thereby accessing messages, emails, social media, GPS, and Uber. It is interesting to note the specific mention of the iPhone, which has emerged as an elite and popular smartphone symbol. The characters of the novel, Naren, Rohit, Ifra, Gyaan, Cyrus and Amanda’s usage of an iPhone is symbolic of their privileged social status. As the most well-adapted smartphone, it is highly used by immigrants as a tool of DMI, “iPhone is but one project among many that seek to modify the mobile to better take account of the things users expect from Internet and computing cultures” (Goggin, 2009).

Throughout the narrative, we can see that the character of an American immigrant in India is largely dependent on the digital infrastructure. It is through social media that Amanda learns about “Naren Agashe’s one-way ticket to India”. During her flight to India, she holds on to Naren’s iPAd as an object of security and assurance to keep her connected to the land she is leaving behind, “Over the rim of his iPad, her breathing steadies” (Rege 21). Naren’s iPad features in the novel as a window to the outside world. It not only becomes his tool of entertainment and distraction while waiting in the immigration queue at the airport but also validates his return back to his country with news and posts celebrating the landmark election. Once both Amanda and Naren are in India, they explore and re-explore the city of Mumbai through close interaction with the digital platforms. The keyword “Uber” occurs 17 times in the novel as an American multinational transportation service operating in India through the digital platform. The news of her grandmother’s death reaches Amanda through the digital medium on a video call after three emails and several missed calls. The screen of her laptop then becomes the only way out to share the grief of Nana’s death. Laptops or smartphones, as valuable DMI in maintaining contact back home, must be charged regularly. Amanda runs out of charge on her phone, misses urgent calls from home, and hence switches to her laptop. “Three emails, all from home. Please call, it is urgent…Why will not you pick up your phone? Trying to reach you…Nana is gone” (Rege 291). Her return to New England is planned online, where the DMI administers the whole process of return, from booking the tickets and arranging transport to getting updates from home.

The internet has become an increasingly impactful medium for knowing about a person who lives away. Naren’s close relatives learn about his return through social media as he posted it online. As an ambitious American returnee, Naren seeks social media as a medium for updates and validation. His recollection of Amanda fades after her college days only because she does not post online often. The idea of posting their whereabouts is very common with migrants to share their stories, experiences and achievements. The bar graph, Figure 5F depicts the usage of the older forms of infrastructure for the communication of migrants. Here, the keyword PCO becomes a site for reminiscing the childhood days of the quarter-life characters Rohit and Omkar in the novel. Rohit is surprised to spot PCO booths while visiting his Ratnagiri as objects of nostalgia, “STD ISD PCO. Nostalgia for the town’s mediocrity, for childhood brands that he assumed the whole country left behind when the Agashes did” (Rege 138). The keyword “computer” also occurs in the same context, in the shop signs and billboards, comprising the country’s new middle class in a small and old town.

The novel also incorporates the traditional forms of migration infrastructure, such as letters and text messages, without the intervention of the Internet. Figure 5G depicts a comparative occurrence of keywords of the two different modes of communication. Since the novel uses both text and message to refer to text messages on a cell phone, we have considered both keywords. Using letters at every instance aims to serve an official purpose. Synonymous with emails, the characters in the novel interact through letters in a formal office setup noted amongst the staff of Ashray and Naren. Text messages, on the other hand, are traditionally associated with cell phones, which may or may not facilitate digital amenities. However, Rege presents the characters using smartphones, and hence, the messages are communicated through an online platform. Several text messages are exchanged between them, often delivering important information or expressing emotional outbursts for each of the characters. The author vividly brings together the dynamism of audio and video messages, breaking the monotony of constantly gazing at static texts on screen. “She responds with a recorded message of her laughter” (Rege 68). Amanda’s audio message to Rohit connotes DMI’s significance in conveying a message, even mirth, through the recordings, which is otherwise not possible for migrants across borders. Amanda’s homesickness in India flashes in a second with the alert of her mother’s message, making her isolation even more haunting. But at the same time, the message brings her back to the flavor of home, of the quiet mornings with the TV and radio voices, “She is alone in the darkness. There is a message on her phone from Mom, asking how she’s feeling” (Rege 124).

The graph, Figure 5H presents Rege’s direct depiction of the return and migration motifs in *Quarterlife*. Naren offers the very first insights on the nature of immigration in America. As a land brimming with opportunities, America harbors hundreds of immigrant communities. However, racism persists both in its workspaces and in its social structures,

“[I]t’s not like race does not exist. We aren’t brown there the way we are brown in the UK where you find an Indian bus boy or delivery guy because there’s this long history of migration from all classes. Most Indians in America got there in the sixties…they only needed jobs. We’re one of the highest earning ethnic minorities, there’s almost a positive bias” (Rege 80).

Hence, the return of these Indian immigrants became more complicated. Return home is a recurrent motif throughout the novel. Though the idea of home reoccurs several times, the specific return home is important for our analysis. Hence, our selection of the keyword “back home” occurs five times in the novel, mostly in reference to Naren’s return. Return in the context of returning to the homeland, occurring eighteen times, forms the central narrative of Naren and Ifra, as discussed in textual analysis 4.4. Towards the end of the novel, Gyaan’s return to New Delhi also becomes a concern. Though swearing on never returning to his home city, now that Rohit’s venture has failed, he has no option but to leave Mumbai and settle back in the comforts of his own home.

The author presents the predicament of every returnee through Naren, “Before you set out, he thinks, you do not know what you know; when you are out there, you know what you do not know; when you return, you know what you know” (Rege 152). He perceived his return to India to be a glamorous affair. However, the hope soon fades away into grim dismay, where the ideologies of even an American returnee are constantly put into question and countered by the set standards. Out in the elite professional space of the city, it becomes a challenge for him to establish his own identity. And all these he only learns and experiences on his return, otherwise oblivious to a brown with a successful life in a white land.

As a novel written in 2023, *Quarterlife* has the highest engagement of digital infrastructure, with return as one of its core themes. The concurrence analysis thus reveals the manifold paradigms of return and DMI within the three novels. Digital infrastructure facilitates multiple forms of migration, and it is fascinating to understand the differences between DMI intervention for new immigrants and regular returnees.

## 7 Conclusion

“Technology invariably embodies the attitude, prejudice, resistance, moral, ethical, social, and cultural aspects of the period in which it arises, as it is not always deployed as a neutral factor” (Shanmugapriya et al., 2022). The paper hence aims to bridge the gap in literary studies where technology becomes the instrument in interpreting “cultural transmission”. This leads us to the ubiquitousness of technology woven within the time. Technology has pervaded human lives over the ages and the digital medium has only aggravated its usage to a wider scale. It is more of a natural transition of technology that migration in the current age will involve digital devices. Our study highlights the impact of digital infrastructures paving the way for transnational migrations, a literary reflection over the period of 2000–2023.

**Figure 6:**
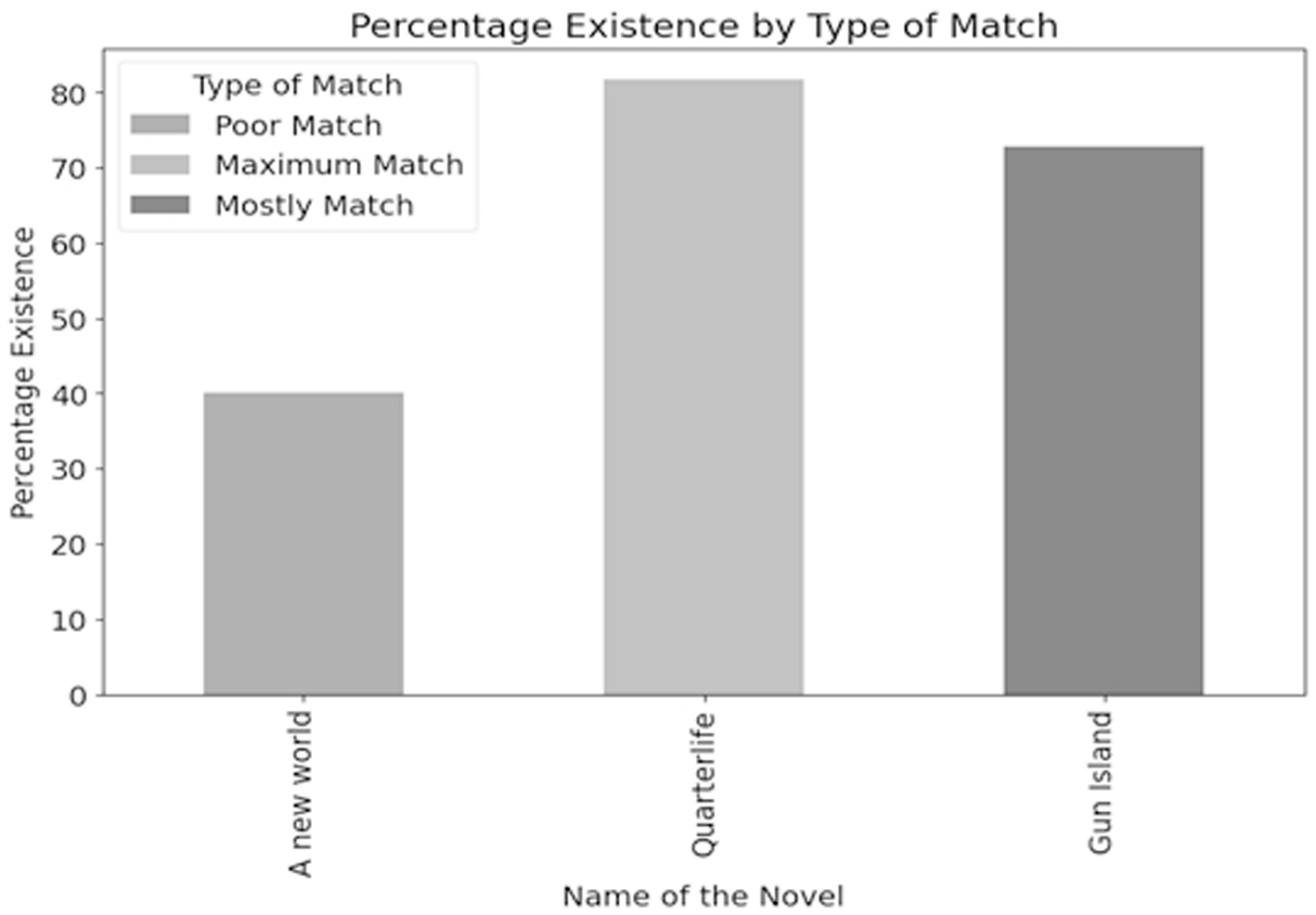
The rising trend of DMI.

The paper traces the patterns of migration and return, representative of contemporary Indian English novels. In the study we investigate how the availability of digital differs in the migratory patterns of a privileged and a basic migrant, in *Gun Island* and *Quarterlife*, between the illegal migrants and the American returnees. The key findings depict the gradual development of digital infrastructure through novels as shown in Figure 6, where the percentage of DMI increases over time. The concurrence analysis reveals the increased interaction of the digital medium in migration of the characters of the three novels, where it is seen that A *New World* with poor match has the least percentage of engagement and *Quarterlife* as the most recent novel has the highest match of the DMI keywords. As a novel written in 2000, it has negligible use of the digital medium, rather is intercepted by the traditional forms of letters, telephones and physical spaces of airlines. Whereas *Gun Island* offers a panoramic representation of the techniques devised to undertake both the regular and illegal forms of migration across borders. The recent DMI and ICT not only occur in the novel but in turn, carry forward the narrative. *Quarterlife* in the same way portrays an active engagement of the digital infrastructure in migration and in maintaining transnational ties, post migration.

Our study makes a significant contribution to the emerging field of digital humanities since it gives an idea of how computational methods, especially *Python*, can bring innovation into the approaches toward literary analysis in ways that traditional approaches alone would not have achieved. It is a state-of-the-art framework for text mining in literary studies, showing exactly how *Python* can be used reliably to analyze textual data for latent structures. As a contribution to the field of digital humanities, the study assimilates computational tools and literary texts to decipher the role of digital infrastructure in migration. The concurrence analysis rooted in text and theory exemplifies interdisciplinarity between the humanities and the computational sciences by combining literary analysis with programming. This research makes new ways of questioning possible and makes explicit a sea change in how digital tools can be incorporated in humanities research, setting up a reference point for future studies in digital humanities. It extends the scope of computational methods to make literary analysis richer and more extensive. As a contribution to both methodology and substance in literary studies, the study also emphasizes automation and reproducibility, which is a characteristic advantage of computational tools. Quantification at the same time allows accounting for the prevalence of underlying themes, thereby adding an objective dimension to a work of literature. Unlike the available text mining tools, the concurrence analysis method here which is considered for development into a web application in near future, proposes to cater to specific needs of the user where the data made available to them comes in handy for the study. The analysis reveals close links between the sociology of literature and return migration. The textual and concurrence analysis together serve as a template to understand the scholarship of return migration, its trends and patterns in India. The paper also examines the rising involvement of the digital infrastructure in tracing migratory routes and connections, continents apart. The selected novels serve as a window to the larger literary landscape of India where return migration emerges as a dominant motif. The interdisciplinary approach hence reflects upon the “enmeshed ripple effect of the technological and the human” that forms the structure of cultural practices like migration^24^, linking the ties of literature as a real-life depiction of social migration through the tools of digital humanities.

## Data Availability

The original contributions presented in the study are included in the article/supplementary material, further inquiries can be directed to the corresponding author.

## Footnotes

^1^Lindquist et al. (2012) introduce the idea of migration infrastructure as black box, a ‘gaping theoretical hole’, where migration infrastructure in itself is not scrutinized for understanding but rather taken for granted.

^2^Harney (1977) conceptualized the migration industry as the ‘commerce of migration’.

^3^Luers and Smet et al. (2018) in “Five questions for digital migration studies.”

^4^The concept that technology is pharmacological was introduced by the philosopher Stiegler (2007) who borrowed it from Derrida and can be traced back to Plato (Venn et al., 2007). ‘Pharmakon’ means both medicine and poison, blessing and bane, which always do not function uniformly for everyone. “Pharmakon is both something which allows us to take care and something to be aware of.”

^5^Digital passages, as propounded by Leurs, “considers the digitization of key identity-formation processes, such as coming of age, rites of passage and the negotiation of offline/online gender, diaspora, religious and youth cultural expectations” (Leurs, 2015).

^6^Habitus as derived from Pierre Bourdieu’s concept refers to “systems of durable, transposable dispositions, structured structures predisposed to function as structuring structures, that is, as principles which generate and organize practices and representations that can be objectively adapted to their outcomes without presupposing a conscious aiming at ends or an express mastery of the operations necessary in order to attain them” (Bourdieu, 1977).

^7^Zijlstra and Liempt (2017) introduced the concept of smartphone traveling focusing on the use of smartphones in the journey of irregular migrants to Europe.

^8^The U-curve theory depends on the level of adjustment on a foreign land, “adjustment as a process over time seems to follow a U-shaped curve: adjustment is felt to be easy and successful to begin with; then follows a ‘crisis’ in which one feels less well adjusted, somewhat lonely and unhappy; finally one begins to feel better adjusted again, becoming more integrated into the foreign community” (Lysgaard, 1995).

^9^“Homelooseness is a more secular experience of mobility which, for many, is now ordinary and even welcome…if rarely chosen entirely without ambivalence” (Wood, 2014).

^10^Baldassar (2001) describes visits home as “a secular pilgrimage of redemption in response to the obligation of child to kin, townsperson to town.”

^11^Morley (2017) proposed the idea of mobile lifestyle to denote “a rich mixture of terrestrial and satellite media consumption, long-distance and local telephony, street gossip, and Internet exchanges alongside regular long-distance travel.”

^12^The names of Bengali dishes as mentioned in the novel, ‘luchi’ meaning flat and round fried bread and ‘mach’ meaning fish in curry form.

^13^Wilton (2021) uses the term ‘butchery metaphor’ to connote the role of governments across the world in “turning a blind eye to the suffering brought about by climate-related loss, as long as it is economically advantageous to do so.”

^14^A frequent circular movement between two or more places.

^15^Chu (2019) defines diaspora visits made by Asian migrants as visits to “home countries to maintain ties with family and friends, to transmit familial and cultural ties to their offspring, tour and vacation.”

^16^ https://scroll.in/article/927202/my-book-is-not-an-apocalyptic-book-at-all-i-guess-im-leaving-hope-as-a-possibility-amitav-ghosh

^17^Devika Rege in conversation with Rege and Sen (2024), in describing the moral, cultural and economic changes from 2014. https://thewire.in/books/not-a-storyteller-an-interview-with-quarterlife-author-devika-rege.

^18^Return of innovation refers to returnees “who are ‘prepared to make use of all the means and new skills they have acquired during their migratory experiences’ with a view to achieving their goals in their origin countries, which, according to them, offer greater opportunities to satisfy their expectations” (Cassarino, 2004).

^19^The Hindi words ‘janmabhoomi’ and ‘karmabhoomi’ mean the land of birth and the land of work or job in English as used by Devika Rege in the novel. “You were ready for the grind. You called Aai on your first night in New York and said, This is my karmabhoomi.” (Rege 75)

^20^Avtar Brah first introduced the concept of ‘homing desire’ for the diaspora migrants who have a home to return back to.“The concept of diaspora places the discourse of ‘home’ and ‘dispersion’ in creative tension, inscribing a homing desire…Not all diasporas inscribe homing desire through a wish to return to a place of ‘origin’” (Brah, 1996).

^21^Cheng (2001) defines the melancholy of race as a “condition that troubles both whites and oppressed minorities: whites are haunted by the memory of minorities whose presence and histories of oppression they overtly deny but covertly remember.”

^22^Data derived from https://www.internetworldstats.com/emarketing.htm.

^23^Walter Benjamin, *Arcade Project* (1999).

^24^Shanmugapriya et al. (2022) cites Goody (2011) to highlight the ripple effect of technology and human, “as the century progressed it became impossible to maintain an absolute distinction between the organic expressions of human nature and the technological process, forms and devices which recorded and communicated those expressions as culture.”

## References

[ref1] Akanle O. (2018). International migration narratives: systematic global politics, irregular and return migration. Int. Soc. Rev. 33, 161–170. doi: 10.1177/0268580918757105

[ref2] Appadurai A. (2015). “Foreword” in Infrastructural lives: Urban infrastructure in Context. eds. Graham S. McFarlane C. (Oxon: Routledge).

[ref3] Arthur N. (2003). Preparing international students for the re-entry transition. Can. J. Couns. 37, 173–185.

[ref4] Baas M. (2015). Transnational migration and Asia: The question of return. Amsterdam: Amsterdam University Press.

[ref5] Baldassar L. (2001). Visits home: Migration experiences between Italy and Australia. Melbourne: Melbourne University Publishing.

[ref6] Baldassar L. Nedelcu M. Merla M. Wilding R. (2016). ICT-based co-presence in transnational families and communities: challenging the premise of face-to-face proximity in sustaining relationships. Global Netw. J. Trans. Aff. 16, 133–144. doi: 10.1111/glob.12108

[ref7] Balibar E. (2004). We, the people of Europe? Reflections on transnational citizenship. Princeton, NJ: Princeton University Press.

[ref8] Bartram D. Poros M. V. Monforte P. (2014). Key concepts in migration. London: Sage.

[ref9] Benjamin W. (2002). Arcades Project. USA: President and Fellows of Harvard College.

[ref10] Bhabha K. H. Klaus S. (2017). Diaspora and home: An interview with Homi K. Bhabha. De Gruyter Conversations. Available at: https://blog.degruyter.com/diaspora-and-home-interview-homi-k-bhabha/

[ref11] Bilgili O. (2022). “Return and transnationalism” in Handbook of return migration. eds. King R. Kuschminder K. (Glos and Massachusetts: Edward Elgar Publishing ltd), 38–52.

[ref12] Bingham L. M. Narvaez S. Perosa T. Aizeki M. (2023). Digital migration control infrastructure as Worldmaking. Thomas Journal of Law and Public Policy: University of St.

[ref13] Bornstein M. H. (2017). The specificity principle in acculturation science. Perspect. Psychol. Sci. 12, 3–45. doi: 10.1177/1745691616655997 28073331 PMC5234695

[ref14] Bourdieu P. (1977). Outline of a theory of practice. Cambridge: Cambridge University Press.

[ref15] Bovenkerk F. (1974). The sociology of return migration: A bibliographic essay. Netherlands: The Hague.

[ref16] Brah A. (1996). Cartographies of diaspora: Contesting identities. London: Routledge.

[ref17] Carames P. A. Suarez F. B. Lama C. A. (2021). “Virtual communities in intra-European mobilities as mechanisms of integration and social exclusion: the new Spanish migration in Europe” in Young people, social inclusion and digitalisation. eds. Moxon D. Serban A. M. Potocnic D. Connoly N. Pasic L. Stefan V. (Cedex: Council of Europe Publishing), 149–161.

[ref18] Cassarino J. P. (2004). Theorising return migration: the conceptual approach to return migrants revisited. Int. J. Multic. Soc. 6, 253–279. https://ssrn.com/abstract=1730637

[ref19] Cerase F. P. (1974). Expectations and reality: a case study of return migration from the United States to southern Italy. Int. Migr. Rev 8, 245–262. 12339170

[ref20] Chaudhuri A. (2000). A New World. London: Picador.

[ref21] Cheng A. A. (2001). The melancholy of race. New York: Oxford University Press.

[ref22] Chu P. P. (2019). Where I have never been: Migration, melancholia, and memory in Asian American narratives of return. Philadelphia: Temple University Press.

[ref23] Cohen R. (1997). Global diasporas: an introduction. New York: Routledge.

[ref24] Constantinides P. Henfridsson O. Parker G. G. (2018). Introduction-platforms and infrastructures in the digital age. Inf. Syst. Res. 29, 381–400. doi: 10.1287/isre.2018.0794

[ref25] Conway D. Robert B. P. (2016). Return migration of the next generations: 21st century transnational mobility. Oxon: Routledge, 2016.

[ref26] Diminescu D. (2008). The connected migrant: an epistemological manifesto. Soc. Sci. Inf. 47, 565–579. doi: 10.1177/0539018408096447

[ref27] Diminescu D. (2020). “Researching the connected migrant” in The sage handbook of media and migration. eds. Smets K. Leurs K. Georgiou M. Witteborn S. Gajjala R. (London: Sage), 74–78.

[ref9002] Düvell F. Preiss C. (2022). “Migration Infrastructures: How Do People Migrate?”, in Introduction to Migration Studies, ed. P. Scholten (Switzerland: Springer, Cham), 83–98.

[ref28] Erdal M. B. (2017). “Timespaces of return migration: the interplay of everyday practices and imaginaries of return in transnational social fields” in Timespace and international migration. eds. Mavroudi E. Page B. Christou Cheltenham A. (UK and Northampton, MA, USA: Edward Elgar Publishing), 104–118.

[ref29] Ghosh A. (2006). The Hungry Tide. London: HarperCollins.

[ref9005] Ghosh A. (2019). Gun Island: A Novel. Gurugram: Penguin Random House.

[ref30] Ghosh A. (2021). The great uprooting: migration and displacement in an age of planetary crisis. Mass. Rev. 62, 712–733. doi: 10.1353/mar.2021.0158

[ref31] Gill H. Ghosh A. (2019). My book is not an apocalyptic book at all. I guess I’m leaving hope as a possibility’: Amitav Ghosh, scroll. Available at: https://scroll.in/article/927202/my-book-is-not-an-apocalyptic-book-at-all-i-guess-im-leaving-hope-as-a-possibility-amitav-ghosh

[ref32] Gmelch G. (1980). Return migration. Annu. Rev. Anthropol. 9, 135–159. doi: 10.1146/annurev.an.09.100180.001031 12264425

[ref33] Goggin G. (2009). Adapting the Mobile phone: the iPhone and its consumption. Continuum 23, 231–244. doi: 10.1080/10304310802710546

[ref34] Goody A. (2011). Technology, literature and culture: Themes in 20th and 21st century literature and culture. Cambridge: Polity Press.

[ref35] Gullahorn J. T. Gullahorn J. (1963). An extension of the U-curve Hypothesis1. J. Soc. Issues 19, 33–47. doi: 10.1111/j.1540-4560.1963.tb00447.x

[ref36] Haas De H. Fokkema T. Fihri M. F. (2014). Return migration as failure or success? The determinants of return migration intentions among Moroccan migrants in Europe. Int. Migrat. Integ. 16, 415–429. doi: 10.1007/s12134-014-0344-6 PMC4486414 26161043

[ref37] Hannam K. Sheller M. Urry J. (2006). Editorial: Mobilities, Immobilities and moorings. Mobilities 1, 1–22. doi: 10.1080/17450100500489189

[ref38] Harney R. F. (1977). The commerce of migration. Can. Ethn. Stud. 9, 42–53.

[ref39] Hooks B. (1989). Choosing the margin as a space of radical openness. Framework J. Cinema Media 36, 15–23.

[ref40] Horvat T. Havas L. Logozar R. (2015). The analysis of keyword occurrences within specific parts of multiple articles — the concept and the first implementation. Tech. J. 8, 362–369. https://hrcak.srce.hr/131563

[ref41] Hron M. (2009). Translate pain: Immigrant suffering in literature and culture. Toronto: University of Toronto Press.

[ref42] Jain P. C. (1982). Indians abroad: a current population estimate. Econ. Polit. Wkly. 17, 299–304.

[ref43] Jain P. C. (1989). Emigration and settlement of Indians abroad. Sociol. Bull. 38, 155–168.

[ref44] Kaur R. (2020). Gun Island by Amitav Ghosh. Mod. Lang. Stud. 50, 94–95.

[ref45] King R. Katie K. (2022). Handbook of return migration. Glos and Massachusetts: Edward Elgar Publishing ltd.

[ref46] Kunuroglu F. Vijver F. Yagmur K. (2016). Return migration. Online Read. Psychol. Culture 8:2. doi: 10.9707/2307-0919.1143

[ref47] Larkin B. (2013). The politics and poetics of infrastructure. Annu. Rev. Anthropol. 42, 327–343. doi: 10.1146/annurev-anthro-092412-155522

[ref48] Latonero M. Kift P. (2018). On digital passages and Borders: refugees and the new infrastructure for movement and control. Soc. Media Soc. 4:1. doi: 10.1177/2056305118764432

[ref49] Leon R. H. (2013). “Conceptualizing the migration industry” in The migration industry and the commercialization of international migration. eds. Hansen T. G. Sorensen N. N. (Oxon: Routledge), 24–44.

[ref50] Leurs K. (2015). Digital passages: Migrant youth 2.0: Diaspora, gender and youth cultural intersections. Amsterdam: Amsterdam University Press.

[ref51] Leurs K. (2020). “Migration infrastructures” in The Sage Handbook of Media and Migration. eds. Smets K. Leurs K. Georgiou M. Witteborn S. Gajjala R. (London: Sage), 91–102.

[ref52] Leurs K. Patterson S. (2020). “Smartphones: digital infrastructures of the displaced” in The handbook of displacement. eds. Adey P. Bowstead J. C. Brickell K. Desai V. Dolton M. Pinkerton A. (Switzerland: Palgrave Macmillan), 583–597.

[ref53] Leurs K. Smets K. (2018). Five questions for digital migration studies: learning from digital connectivity and forced migration in(to) Europe. Soc. Media Soc. 4:1. doi: 10.1177/2056305118764425

[ref54] Lindquist J. Xiang B. Yeoh B. S. A. (2012). Introduction: opening the black box of migration: brokers, the Organization of Transnational Mobility and the changing political economy in Asia. Pac. Aff. 85, 7–19. doi: 10.5509/20128517

[ref56] Loomba A. Kaul S. (1994). Introduction: Location, culture. Oxford Literary: Post-Coloniality Review.16:1/2, 3–30.

[ref57] Lysgaard S. (1995). Adjustment in a foreign society: Norwegian Fulbright grantees visiting the United States. Int. Soc. Sci. Bull. 7, 45–50.

[ref58] Madhavan M. C. (1985). Indian emigrants: numbers, characteristics, and economic impact. Popul. Dev. Rev. 11, 457–481. doi: 10.2307/1973248

[ref59] Martin B. Mohanty C. D. (1986). “Feminist politics: What’s home got to do with it? Feminist studies/critical studies” in Language, discourse, society. ed. Lauretis T. (London: Palgrave Macmillan), 191–212.

[ref60] Menon N. (2016). Remapping the Indian postcolonial canon: Remap, reimagine and retranslate. London: Palgrave Macmillan.

[ref61] Mille A. (2013). Des traces à l’ère du web. Intellectica 59, 7–28.

[ref62] Miller S. (2023). Migrants: the story of us all. London: Abacus.

[ref63] Morley D. (2017). Communications and mobility: the migrant, the Mobile phone and the container box. NJ: Wiley Blackwell.

[ref64] Mukherjee U. (2010). Postcolonial environments nature, culture and the contemporary Indian novel in English. UK: Palgrave Macmillan.

[ref65] Mukherjee B. (2011). Immigrant writing: changing the contours of a National Literature. Am. Lit. Hist. 23, 680–696. doi: 10.1093/alh/ajr027

[ref66] Mukherjee N. (2015). Odysseus abroad by Amit Chaudhuri review – audaciously redraws the modernist map. The Guardian. Available at: https://www.theguardian.com/books/2015/feb/07/odysseus-abroad-amit-chaudhuri-review-audaciously-redraws-modernist-map

[ref67] Parella S. Petroff A. (2019). Return intentions of Bolivian migrants during the Spanish economic crisis: the interplay of macro-Meso and micro factors. J. Int. Migr. Integr. 20, 291–305. doi: 10.1007/s12134-018-0607-8

[ref68] Parella S. Petroff A. Speroni T. Piqueras C. (2019). Social suffering and return migrations: a conceptual proposal. Apuntes 84, 33–57. doi: 10.21678/apuntes.84.1013

[ref69] Press C. (2022). “Digital migration infrastructures” in Introduction to migration studies: Interactive guide to the literature on migration and diversity. ed. Scholten P. (Netherlands: Springer), 99–109.

[ref70] Rege D. (2023). Quarterlife: a novel. Gurugram: HarperCollins.

[ref71] Rege D. Sen J. (2024). Not a storyteller: An interview with 'Quarterlife' author Devika Rege. The Wire. Available at: https://thewire.in/books/not-a-storyteller-an-interview-with-quarterlife-author-devika-rege

[ref72] Sam D. L. Berry J. W. (2010). Acculturation: when individuals and groups of different cultural backgrounds meet. Perspect. Psychol. Sci. 5, 472–481. doi: 10.1177/1745691610373075 26162193

[ref73] Sayad A. (2018). The suffering of the immigrant. Germany: Polity Press.

[ref74] Shanmugapriya T. Menon N. Sutton D. (2022). “Quantitative stepwise analysis of the impact of Technology in Indian English Novels 1947–2017” in Literary cultures and digital humanities in India. eds. Zaidi N. Pue A. S. (London: Routledge), 206–224.

[ref75] Smith A. (2004). “Migrancy, hybridity and postcolonial literary studies” in The Cambridge companion to postcolonial literary studies. ed. Lazarus N. (Cambridge: Cambridge University Press), 241–261.

[ref76] Sonnenschein K. Michelini C. King B. (2021). Betwixt and between a qualitative review of the (re)acculturation of international students and returnees. British J. Guid. Counsell. 51, 17–28. doi: 10.1080/03069885.2021.1998884

[ref77] Spivak G. C. Sara H. (1990). The post-colonial critic interviews, strategies, dialogues. New York and London: Routledge.

[ref78] Stefansson A. H. (2004). “Homecoming to the future: form diasporic Mythographies to social projects of return homecoming: unsettling paths of return” in Homecoming: Unsettling paths of return. eds. Markowitz F. Stefansson A. H. (Maryland: Lexington Books), 2–20.

[ref79] Stiegler B. (2007). Questions de pharmacologie générale. Il n’y a pas de simple pharmakon. Psychotropes 13, 27–54.

[ref80] Sussman N. M. (2010). Return migration and identity: A global phenomenon, a Hong Kong case. Hong Kong: Hong Kong University Press.

[ref81] Thompson R. G. (2014) Narratives of return: The contemporary Caribbean woman writer and the quest for home. [PhD dissertation]. [Goldsmith College]: University of London. Available at: https://research.gold.ac.uk/id/eprint/11741/

[ref82] Tsuda T. (2009). Diasporic homecoming: Ethnic return migration in comparative perspective. Stanford: Stanford University Press.

[ref84] Vathi Z. Russell K. (2017). Return migration and psychological wellbeing: discourses, policy-making and outcomes for migrants and their families. New York: Routledge.

[ref85] Venn C. Boyne R. Phillips W. P. Bishop R. (2007). Technics, media, teleology: interview with Bernard Stiegler. Theory Cult. Soc. 24, 334–341. doi: 10.1177/0263276407086403

[ref86] Wilton D. (2021). We are the dispossessed’: displacement, knowledge production and bare life in West Bengal climate fiction. Parallax 27, 344–361. doi: 10.1080/13534645.2022.2071249

[ref87] Wood J. (2014). On not going home. London Review of Books. 36:4. Available at: https://www.lrb.co.uk/the-paper/v36/n04

[ref88] Xiang B. Lindquist J. (2014). Migration infrastructure. Int. Migr. Rev. 48, 122–148. doi: 10.1111/imre.12141

[ref89] Young R. C. (1995). Colonial desire: Hybridity in theory, culture and race. New York: Routledge.

[ref9001] Young R. C. (2012). Postcolonial remains. New Literary History, 43, 19–42. doi: 10.1353/nlh.2012.0009

[ref90] Young R. J. (2017a). Postcolonial remains. New Lit. History 43, 19–42. doi: 10.1353/nlh.2012.0009

[ref91] Young R. J. (2017b). The Dislocations of Cultural Translation. PMLA 132, 186–197. doi: 10.1632/pmla.2017.132.1.186

[ref92] Zijlstra J. Liempt I. V. (2017). Smart(phone) travelling: understanding the use and impact of mobile technology on irregular migration journeys. Int. J. Mig. Border Stud. 3:174. doi: 10.1504/IJMBS.2017.083245

